# Duodenal transcriptomics demonstrates signatures of tissue inflammation and immune cell infiltration in children with environmental enteric dysfunction across global centers

**DOI:** 10.1016/j.ajcnut.2024.02.023

**Published:** 2024-09-17

**Authors:** Chelsea Marie, Subhasish Das, David Coomes, Tahmeed Ahmed, S Asad Ali, Junaid Iqbal, Paul Kelly, Mustafa Mahfuz, Sean R Moore, William A Petri, Phillip I Tarr, Lee A Denson, EEDBI Consortium, Kumail Ahmed, Sheraz Ahmed, Md Ashraful Alam, David Auble, SM Khodeza Nahar Begum, Ellen Besa, Mubanga Chama, Donna M Denno, Shah Mohammad Fahim, Md Amran Gazi, Yael Haberman, Rashidul Haque, Md Mehedi Hasan, Md Shabab Hossain, Aneeta Hotwani, Najeeha Talat Iqbal, Ning-Jiun Jan, Furqan Kabir, Pankaj Kumar, Ta-Chiang Liu, Barbara J Mann, Ramendra Nath Mazumder, Anwaruddin Mohammad, Christopher A Moskaluk, Uma Nayak, Malick Ndao, Shyam S Ragahavan, Masudur Rahman, Najeeb Rahman, Kamran Sadiq, Shafiqul Alam Sarker, Nurmohammad Shaikh, Peter B Sullivan, Guillermo J Tearney, Fayaz Umrani, Omer H Yilmaz, Kanekwa Zyambo

**Affiliations:** 9 Department of Paediatrics and Child Health, Aga Khan University, Karachi, Pakistan; 10 Nutrition Research Division, International Centre for Diarrhoeal Disease Research, Bangladesh, Dhaka, Bangladesh; 11 Department of Biochemistry and Molecular Genetics, University of Virginia School of Medicine, Charlottesville, Virginia, USA; 12 Department of Pathology, Bangladesh Specialized Hospital, Dhaka, Bangladesh; 13 Tropical Gastroenterology & Nutrition Group, University of Zambia School of Medicine, Lusaka, Zambia; 14 Department of Pediatrics, University of Washington School of Medicine, Seattle, WA, USA; 15 Division of Pediatric Gastroenterology, Hepatology, and Nutrition, Cincinnati Children’s Hospital Medical Center, Cincinnati, OH, USA; 16 Department of Medicine, University of Virginia School of Medicine, Charlottesville, Virginia, USA; 17 Department of Pathology and Immunology, Washington University School of Medicine, St. Louis, MO, USA; 18 Department of Medicine, University of Virginia School of Medicine, Charlottesville, Virginia, USA; 19 Department of Pathology, University of Virginia School of Medicine, Charlottesville, VA, USA; 20 Department of Public Health Sciences, University of Virginia School of Medicine, Charlottesville, Virginia, USA; 21 Department of Pediatrics, Washington University School of Medicine, St. Louis, MO, USA; 22 Department of Gastroenterology, Sheikh Russel National Gastroliver Institute and Hospital, Dhaka, Bangladesh; 23 Department of Paediatrics, Children's Hospital, University of Oxford, Oxford, UK; 24 Department of Pathology, Harvard Medical School, Boston, MA, USA; 25 Department of Pathology, Massachusetts General Hospital, Boston, MA, USA; 1 Department of Medicine, University of Virginia School of Medicine, Charlottesville, VA, United States; 2 Nutrition Research Division, International Centre for Diarrhoeal Disease Research, Bangladesh, Dhaka, Bangladesh; 3 Department of Epidemiology, University of Washington School of Public Health, Seattle, WA, United States; 4 Department of Paediatrics and Child Health, Aga Khan University, Karachi, Pakistan; 5 Blizard Institute, Barts and The London School of Medicine, Queen Mary University of London, London, United Kingdom; 6 Department of Pediatrics, University of Virginia School of Medicine, Charlottesville, VA, United States; 7 Department of Pediatrics, Washington University School of Medicine, St. Louis, MO, United States; 8 Division of Gastroenterology, Hepatology and Nutrition, Cincinnati Children's Hospital Medical Center, Cincinnati, OH, United States

**Keywords:** environmental enteric dysfunction, environmental enteropathy, RNA-sequencing, small intestine, WGCNA, biopsy

## Abstract

**Background:**

Environmental enteric dysfunction (EED) is an inflammatory condition of the small intestine that is prevalent in children residing in low- and middle-income countries. EED is accompanied by profound histopathologic changes in the small bowel, loss of absorptive capacity, increased intestinal permeability, increased microbial translocation, and nutrient loss.

**Objectives:**

We sought to identify dysregulated genes and pathways that might underlie pediatric EED.

**Methods:**

RNA-sequencing libraries were generated from endoscopically obtained duodenal tissue from undernourished children with EED from 3 prospective cohorts of children with EED. The EED transcriptome was defined in comparison to North American children without EED. Weighted gene coexpression network analysis (WGCNA) was tested for gene modules associated with EED and its histologic features.

**Results:**

The 1784 upregulated genes in EED were highly enriched for immune and inflammatory processes, including IL-17 and JAK-STAT signaling, and cytokine–cytokine receptor interactions. The 1388 downregulated genes included genes corresponding to xenobiotic metabolism, detoxification, and antioxidant capacities. A gene coexpression module enriched for antimicrobial responses and chemokine activity was significantly associated with villous blunting, goblet cell depletion, and overall histologic severity of EED.

**Conclusions:**

The transcriptome signatures of EED include specific innate and adaptive immune responses that are consistently elevated across study centers, coupled with reduced detoxification and antioxidant capacities. These data may have implications for targeted interventions to improve EED outcomes.

## Introduction

Environmental enteric dysfunction (EED) is an inflammatory condition of the small intestine that is highly prevalent in children and adults in low- and middle-income countries. Small bowel histopathology includes villus blunting and fusion, increased intraepithelial lymphocyte (IEL) infiltration, reduced goblet cell density, and decreased or abnormal Paneth cells (PCs) [1]. These changes have been associated with loss of intestinal absorptive capacity, increased gut permeability and microbial translocation, and nutrient loss [2,3]. EED is present early in life with >80% of children having evidence of intestinal inflammation by 12 wk of age in a prospective birth cohort in Bangladesh [4]. EED is associated with oral vaccine failure, impaired cognitive development, and reduced growth attainment in children [[4], [5], [6], [7]].

Transcriptomics analysis of small bowel tissue in children from Pakistan with EED identified suppression of antioxidant, detoxification, and lipid metabolism genes, and induction of antimicrobial response, interferon, and lymphocyte activation genes in EED. Genes that were upregulated in EED included the inflammatory mediator interferon γ (*IFNG),* the bacterial sensor dual oxidase 2 (*DUOX2*), and dual oxidase maturation factor 2 (*DUOXA2*), which form a heterodimeric NADPH oxidase complex, the antiviral interferon-induced transmembrane (*IFITM*), gene family, and lipocalin 2 (*LCN2*). A parallel epigenetic analysis found that transcriptional regulation was accompanied by hypermethylation of repressed genes and hypomethylation of induced genes in children with EED [8]. Transcriptional profiling of small intestinal biopsies from hospitalized Zambian children with enteropathy associated with severe acute malnutrition also identified alterations in nutrient transporters and increased expression of inflammatory genes correlated with the severity of histopathology [9]. Further analysis of duodenal gene expression profiles in a community-based study of Zambian children with undernutrition correlated markers of microbial translocation and genes involved in barrier function, including mucin 4 (*MUC4*), mucin 13 (*MUC13*), mucin 17 (*MUC17*), and claudin 4 (*CLDN4*) [2].

Understanding the causes and consequences of EED is challenged by the limited ability to assess small intestinal tissue in affected children. Here, we leveraged RNA-sequencing data from the community-based Zambian and Pakistani EED cohorts along with data from a parallel study in Bangladeshi children to define the mucosal profile of EED transcriptomic dysregulation across global sites. The goal of this work was to test the hypothesis that EED induces similar mucosal gene expression patterns across environments, and to relate EED gene expression to the recently developed EED histopathologic scoring criteria [1,10].

## Methods

We collected and analyzed data from 3 prospective community-based cohort studies of EED [Biomarkers of Environmental Enteropathy in Children (BEECH), Bangladesh Environmental Enteric Dysfunction (BEED), and Study of Environmental Enteropathy and Malnutrition (SEEM)]. The details of the collaborators fo the EEDBI Consortium are provided in Appendix B.

BEECH, BEED, and SEEM were conducted in periurban Lusaka, Zambia, urban Dhaka, Bangladesh, and rural Sindh province, Pakistan, respectively. BEED and SEEM included comparison cohorts of children without EED undergoing clinically indicated endoscopy at the University of Virginia Children’s Hospital (UVA) and Cincinnati Children’s Hospital Medical Center (CCHMC), respectively. Signed informed consent from legal guardians was obtained, and all study centers obtained approval for these studies from their respective review boards. Ethical approval was obtained from AKU Ethics Review Committee (ERC) (3836-Ped-ERC-15), icddr,b ERC (PR-16007), University of Zambia Biomedical Research Ethics Committee (006-02-16) and National Health Research Authority (MH/101/23/10/1), UVa Institutional Review Board (19466), CCHMC Institutional Review Board (2016-0387). Exemption was received from the University of Washington Institutional Review Board (IRB) (STUDY00013442) and the Washington University IRB (201801207).

### Study participants and sample size

Each EED site enrolled a large study group for nutritional intervention, and children with insufficient growth following the intervention were invited to be evaluated by endoscopy and biopsies. The first 30–50 children undergoing endoscopy from each EED site had 1–2 biopsies designated for RNA-sequencing (RNA-seq), and transcriptomic data quality checks were applied (see Transcriptomic data processing methods) to arrive at the final sample size (by the site) of BEECH *n* = 28 subjects, BEED *n* = 39 subjects, and SEEM *n* = 49 subjects (Supplemental Figure 1 and Supplemental Table 1). The comparison group was enrolled at 2 North American medical centers (UVA and CCHMC) and consisted of children presenting for endoscopic intestinal biopsy for diagnostic purposes. Children with diagnostic histology consistent with esophagogastrointestinal disease were excluded from the analysis, whereas the nondiagnostic group, comprising those without medical diagnoses or diagnostic histology consistent with esophagogastrointestinal disease were designated as the non-EED comparison group for the transcriptomic analysis (CCHMC *n* = 23 and UVA *n* = 16). Detailed descriptions of each center’s study design, setting, and eligibility criteria have been published [2,11,12] and are summarized in the Supplemental methods and the companion article in this issue [13]. Analyses of RNA samples collected by BEECH and SEEM have been previously published in distinct, single-site studies [2,8].

### Duodenal biopsy sample collection and RNA extraction and sequencing

Detailed methods for each center are provided in the Supplemental methods and summarized in Supplemental Table 1.

### Transcriptomic data processing

FASTQ files from each sequencing center were uploaded to Synapse for central processing. Raw sequencing reads were aligned to the reference genome using the STAR read aligner, and gene counts were quantified, as described previously [14] (Supplemental Figure 2). Gene expression (Y chromosome encoded genes and X-inactive specific transcript (*XIST*) determined sex and the clinically reported sex were examined and 4 miscoded samples were excluded (2 each from BEECH and BEED) from the analysis. Two additional samples from BEECH were excluded due to missing clinical data. BEECH collected replicate biopsies for RNA-seq, thus each set of 2 biopsies excluded corresponds to a single study participant.

### Differential expression analysis

Genes differentially expressed between EED and comparison groups were selected using an absolute value of log_2_ fold-change (LFC) ≥1 and a Bonferroni adjusted *P* value (*P*_adj_) < 0.05 using the R package DESeq2 [15]. Sex, sequencing site, and percent intronic bases were included in the differential expression model based on multivariate regression for the optimal model using sageseqr [16]. We performed principal component analysis to summarize variation in gene expression and visualize the effect of technical and biological covariates between participants (Supplemental Figure 3). BEECH samples clustered separately from other samples, and a sensitivity analysis with and without BEECH was performed as described in the Supplemental material (Supplemental Figure 4). Additionally, site-specific differential expression was performed by comparing each EED site to the joint North American comparison group (Supplemental Figure 5). LFC shrinkage was applied to differential expression gene sets for visualizing and ranking genes [17]. All analyses were performed in R version 4.2.1.

### Gene set enrichment analysis

Biotypes were extracted from biomart annotations [18]. For simplicity, the pseudogene category includes polymorphic, transcribed, processed, and unprocessed pseudogenes. Gene set enrichment analysis (GSEA) was performed using the clusterProfiler 4.0 package [19]. Gene ontology, and Wiki and Kyoto Encyclopedia of Genes and Genomes pathway enrichment were analyzed with a false discovery rate threshold of 0.01. *Q* values were calculated using the Bonferroni correction. Normalized enrichment scores (NES) consider the magnitude of differential expression of genes within the gene set list and represent pathway activation (positive NES) or suppression (negative NES) in EED.

### Weighted gene coexpression network analysis

Signed weighted gene coexpression network analysis (WGCNA) was performed to identify modules of coexpressed genes [20]. For each module in WGCNA, the first principal component, termed the eigengene, summarizes and represents the expression profiles of all the genes in a module. Gene modules associated with EED were identified by correlation analysis between each module eigengenes and an EED diagnosis. Further correlation between gene module eigengenes and histologic parameters (Supplemental Table 2) was performed within the EED group. Modules are referred to by their hub genes. Hub genes are the most highly connected genes within a module and are defined as genes with the highest eigengene-based connectivity score (kME) in the module. Functional enrichment of gene modules was performed using ToppGene suite tools [21] with a *P* value threshold of 0.01. *Q* values were calculated with the Bonferroni correction and the 10 most significant categories are included in the Supplemental Data set file.

### Network analysis

A protein–protein interaction network for the *LCN2* gene module was constructed using the STRING application (V2.0.2) [22] in cytoscape (V3.10.1) [23]. The input was filtered for significantly differentially expressed genes (*P*_adj_ < 0.05) and a stringent filter (STRING score = 0.70) was used to select the largest high-confidence interacting subnetwork. This subnetwork was mapped with differential expression values, and functional enrichment of the protein network was performed using the STRING function within cytoscape.

### *Quantitative small intestinal EED histopathology scores*

The histopathologic scoring criteria are described in a companion manuscript [10]. The total score percent-5 (TSP-5) was calculated from the five most informative histology parameters for differentiating EED from a reference group of American children. These were villus architecture (VA), intraepithelial lymphocytes (IEL), goblet cells (GC), Paneth cells (PC), and intramucosal Brunner’s glands (BG). Higher scores reflect increasing histopathologic severity assessed as increased deviation from the reference group such that higher BG and IEL scores reflect increased density in EED, while higher GC and PC scores reflect lower density in EED, and higher villus architecture scores reflect the increased villus blunting of EED. The scoring criteria and parameters are detailed in Supplemental Table 2. The relationships between quantitative histologic scores and RNA-seq transcript counts were analyzed using a linear mixed-effects model in the R package lme4 (1.1-31) [24] with study center or disease as a fixed effect and patient ID as a random effect to account for replicate measures of gene expression in the BEECH study. RNA counts were log transformed to improve model fit and reduce the influence of outliers.

### Quantitative immunohistochemistry of small intestinal biopsies

Immunohistochemistry (IHC) was performed as described in a companion paper in this supplement [25]. Quantitative IHC values were normalized to either total epithelial area for CD3, CD19, CD45 or total surface area for C-X-C motif chemokine ligand 10 (Cxcl10), granzyme B (Gzmb), Lcn2, defensin alpha 5 (Def5a), mucin 2 (Muc2), regenerating islet-derived 1 beta (Reg1b), solute carrier family 15 member 1 (Slc15a1), and Duox2. Normalization factors were selected based on the recommendation of the lead pathologist (CAM). The associations between quantitative immunohistologic scores and RNA-seq transcript counts were measured using a linear mixed-effects model with the study center as a fixed effect and patient ID as a random effect to account for replicate measures of gene expression in the BEECH study. Both IHC and RNA measurements were log transformed before model fit.

## Results

### Participants

RNA-seq data were obtained from 115 children with EED [BEECH (*n* = 27), BEED (*n* = 39), and SEEM (*n* = 49)], and 39 North American children [UVA (*n* = 16) and CCHMC (*n* = 23)] (Table 1). Differences in anthropometry were present across the EED centers due to different enrollment criteria [13]. The BEED cohort had slightly greater average height-for-age, weight-for-age, and weight-for-height *z*-scores. Children in the North American comparison group were older and not undernourisehd based on BMI (kg/m^2^)), weight-for-height, weight-for-age, or height-for-age.

**TABLE 1:** Participant information

	BEECH *N* = 27	BEED *N* = 39	SEEM *N* = 49	EED combined *N* = 115	CCHMC *N* = 23	UVA = 16	United States combined *N* = 39
Age in y^1^, mean (SD)	1.4 (0.4)	1.6 (0.2)	1.6 (0.3)	1.6 (0.3)	5.7 (2.2)	11.8 (4.1)	8.2 (4.3)
Gender, % F	48.1%	59%	34.7%	46.1%	50%	43.5%	46.2%
HAZ^1^, mean (SD)	−3.5 (0.7)	−2.2 (0.7)	−3.0 (1.1)	−2.8 (1.0)	0.2 (1.0)	0.3 (1.0)	0.2 (1.0)
WAZ^1^^,^^2^, mean (SD)	−2.2 (0.9)	−1.9 (0.8)	−3.1 (0.8)	−2.5 (1.0)	0.2 (1.5)	0.6 (1.3)	0.4 (1.4)
WHZ^1^^,^^3^, mean (SD)	−0.6 (1.0)	−1.2 (0.8)	−2.2 (0.7)	−1.5 (1.1)	-0.2 (1.3)	-	−0.2 (1.3)
BMI^1^^,4^, mean (SD)	—	—	—	—	16.3 (3.9)	20.0 (4.7)	17.8 (4.6)

*Abbreviations*:F, female; HAZ, height-for-age *Z*-score; WAZ, weight-for-age *Z*-score; WHZ, weight-for-height *Z*-score;

1 Measurements closest to biopsy.

2 WHZ can only be calculated for children <5 y. All EED cohorts included children below 5 y. Among United States cohorts, only CCHMC included 8 <5 y.

3 BMI is used as a measure of thinness (or overweight status) among children ≥2 y.

Small intestinal histopathology was evaluated across centers by study pathologists (Table 2). Goblet cell depletion was more severe in BEED than other cohorts, Paneth cell (PC) depletion was less severe in SEEM than in BEECH or BEED, and IELs were less numerous in BEECH than in SEEM or BEED. The total score percent (TSP5), which is an index score which incorporates the 5 most discriminatory histologic features to identify EED and EED severity [10], was similar across EED cohorts, with some center-specific variations. Scores between the 2 North American comparison groups were similar.

**TABLE 2:** Histopathologic EED scores

	Mean (SD) % unscorable						
Feature (scale)	BEECH *N* = 27	BEED *N* = 39	SEEM *N* = 49	EED combined *N* = 115	CCHMC *N* = 23	UVA *N* = 16	Non-EED combined *N* = 39
TSP5 (0%–100%)	52.9 (10.5) 3.7%	55.9 (14.1) 30.8%	46.1 (10.2) 0%	50.4 (12.1) 11.3%	12.4 (8.1) 0%	13.7 (7.5) 0%	13.0 (7.8) 0%
Goblet cell score (0–4)	1.3(0.6) 0%	2.2(0.7) 0%	1.1(0.6) 0%	1.5 (0.8) 0%	0.1 (0.2) 0%	0.4 (0.5) 0%	0.2 (0.4) 0%
Intramucosal Brunner glands (0–3)	0.2 (0.5) 0%	0.6 (0.9) 2.6%	0.5 (0.7) 0%	0.5 (0.7) 0.9%	1.8 (1.2) 0%	2.3 (1.2) 0%	2.0 (1.2) 0%
IEL (0–4)	0.8 (0.6) 0%	1.6 (1.0) 0%	1.9 (0.8) 0%	1.5 (0.9) 0%	0.4 (0.4) 0%	0.3 (0.3) 0%	0.3 (0.4) 0%
Paneth cell score (0–3)	2.4 (0.8) 7.4%	2.2 (1.2) 51.3%	0.8 (0.6) 2%	1.5 (1.1) 20%	0.2 (0.3) 0%	0.5 (0.4) 0%	0.3 (0.4) 0%
Villus architecture (0–4)	2.4 (1.0) 7.4%	2.0 (1.2) 35.9%	2.1 (1.1) 10.2%	2.2 (1.1) 18.3%	0.3 (0.4) 0%	0.6 (0.5) 18.8%	0.4 (0.5) 7.7%

Abbreviations: IEL, intraepithelial lymphocytes; NS, not scorable; TSP5, total score percent top 5.

### Core EED duodenal transcriptome

**FIGURE 1:**
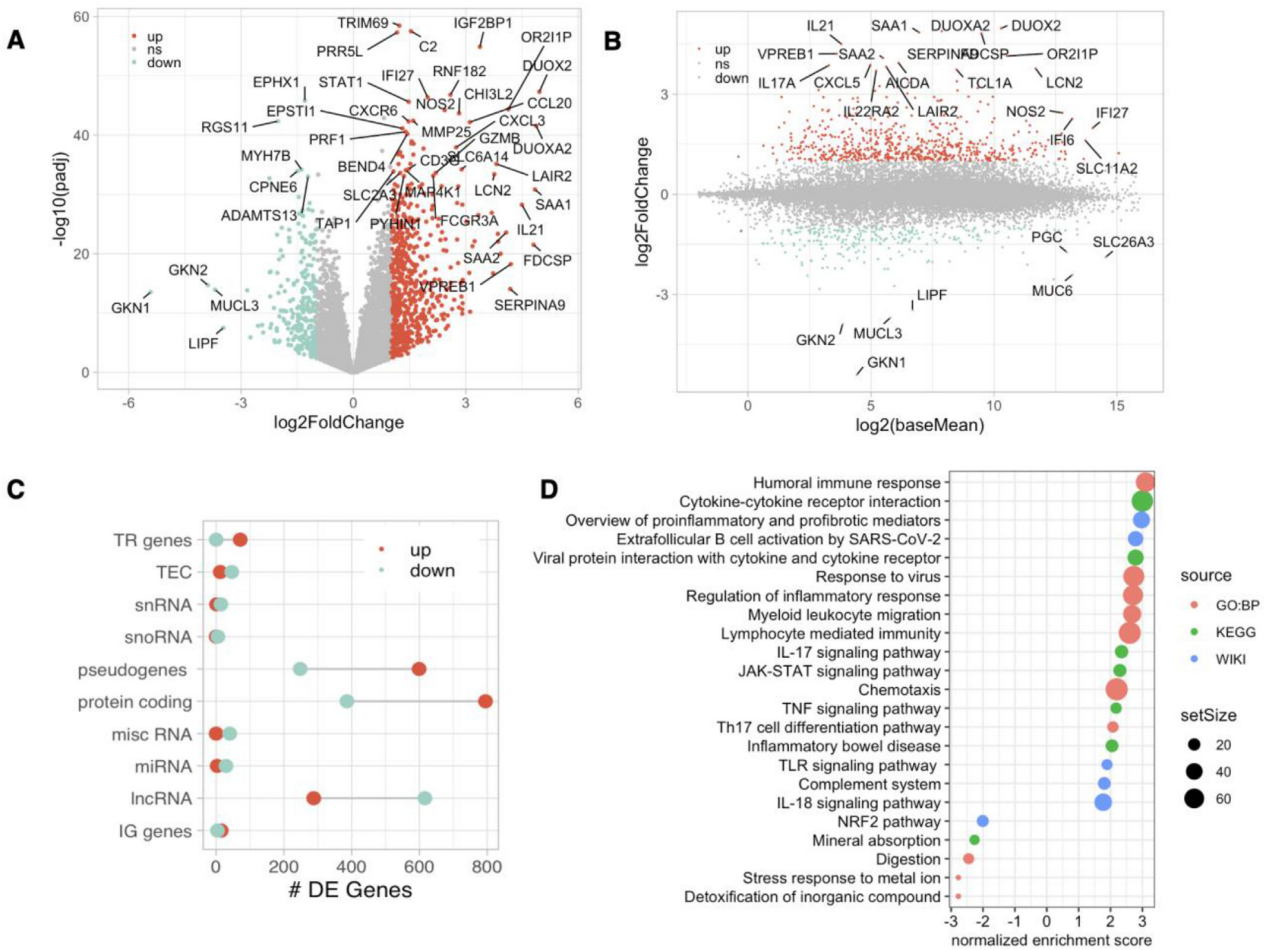
**The EED transcriptome**. (A) Volcano plot of differentially expressed genes in EED. Downregulated, upregulated, and nonsignificant genes are shown in green, red, and gray plots, respectively. Genes are plotted according to the LFC (*X*-axis) and the -log10 (*P*_adj_) (*Y*-axis). (B) Bland–Altman plot showing overall expression (*X*-axis) relative to LFC (*Y*-axis) in EED. (C) The number of differentially expressed genes corresponding to specific biotypes in EED. Upregulated genes in EED are shown in red, downregulated genes are shown in green. TR, T-cell receptor; IG, immunoglobulin. (D) Gene set enrichment of up and downregulated genesets in EED. Enriched categories (*Y*-axis) colored by source and normalized enrichment scores (*X*-axis) are shown.

We defined the core EED duodenal transcriptome as differentially regulated genes in children with EED compared with the North American comparison groups. This analysis identified 3172 differentially expressed genes (Figure 1A, Supplemental Data set 1); 1388 genes were downregulated, and 1784 genes were upregulated in EED. The most highly upregulated genes in EED were the Ig heavy constant delta (*IGHD*) gene (LFC = 5.41 and *P*_adj_ *=* 9.38E-41) and the gene encoding follicular dendritic cell-secreted protein (*FDCSP*) (LFC = 5.16 and *P*_adj_ *=* 3.06E-22), both involved in B-cell maturation. *IGHD* encodes the constant region of the heavy chain of IgD, the major antigen receptor isotype on the surface of peripheral B cells and a marker of naïve B cells [26]. *FDCSP* encodes a follicular dendritic cell-secreted protein, which binds B cells and regulates antibody response to systemic immunization [27] and mucosal IgA production [28]. Serpin family A member 9 (*SERPINA9),* a marker of germinal center B cells [26] was also highly upregulated in EED (LFC = 4.6 and *P*_adj_ *=* 8.95E-15). *DUOX2* (LFC = 5.12 and *P*_adj_ *=* 4.97E-48) and its chaperone, *DUOXA2* (LFC = 5.06 and *P*_adj_ *=* 2.74E-42) were the third and fifth most upregulated genes in EED, respectively. Together, they encode an NADPH oxidase implicated in small bowel epithelial antimicrobial responses [29].

Other highly upregulated genes in EED include serum amyloid A1 and A2 (*SAA1*) and (*SAA2*), which encode acute phase proinflammatory and chemotactic proteins, *IL21*, which encodes a potent cytokine regulator of immune cells, including T follicular helper cells, and *IL17A*, which encodes an inflammatory cytokine that can mediate protective innate immunity to pathogens or contribute to the pathogenesis of inflammatory diseases, such as psoriasis and rheumatoid arthritis.

Genes that were both highly upregulated and highly expressed in the duodenum (defined as in the upper quartile of overall expression as measured by mean counts) of children with EED included *LCN2* (LFC = 3.88, *P*_adj_ = 3.8E-34), olfactory receptor family 2 subfamily I member 1 pseudogene (*OR2I1P*) (LFC=4.25, *P*_adj_*=*4.2E-45), and *DUOX2. REG1B* was also significantly upregulated (LFC=2.53, *P*_adj_*=*1.05E-12) and highly expressed.

The most downregulated genes were related to lipid metabolic functions, including gastrokines 1 and 2 (*GKN1*: LFC=-6.25, *P*_adj_= 5.7E-16 and *GKN2:* LFC=-4.21, *P*_adj_= 3.4E-17) and gastric lipase (*LIPF:* LFC=-4.21, *P*_adj_= 1.51E-09). *GKN1* and *GKN2* are mainly expressed in the stomach, and decreased *GKN1* expression has been linked to gastric cancer [[30], [31], [32]]. Similarly, *LIPF* encodes gastric lipase F, a critical triglyceride metabolic enzyme secreted mainly by chief cells in the gastric fundus [33], the repression of which is associated with gastric cancer [34]. Although *LIPF*, *GKN1*, and *GKN2* were highly repressed, their overall expression is low relative to other duodenal genes (Figure 1B).

Other significantly downregulated genes in EED included mucin 6 (*MUC6*) (LFC=-2.99, *P*_adj_*=*1.1E-8), progastricsin (*PGC*) (LFC=−2.4, *P*_adj_*=2.2E-5*), and solute carrier family 26 member A3 (*SLC26A3*) (LFC=−2.0*, P*_adj_*=*2.1E-22) (Figure 1B). *MUC6* encodes a mucin with protective functions in the duodenum and can be produced by Brunner's glands [35]. Progastricsin is an aspartic proteinase expressed mainly in the gastric mucosa. Circulating progastricsin concentrations are elevated in some stomach disorders, including *Helicobacter pylori* gastritis [36] and colorectal and gastric cancer [37,38]. *SLC26A3* encodes a key intestinal chloride anion exchanger and is a susceptibility gene for inflammatory bowel disease [39]. Reduced expression of *SLC26A3*, in conjunction with increased tumor necrosis factor (*TNF*), compromises gut barrier function [40]. Other significantly downregulated mucins in EED were mucin-like 3 (*MUCL3*) (LFC=−4.01, *P*_adj_*=*1.1E-14), a marker of surface mucosal cells [41], and mucin 5AC (*MUC5AC*) (LFC=-3.6, *P*_adj_*=*1.3E-6)*.* The number of up- and downregulated genes in EED were similar, but the proportion of protein-coding genes was higher for upregulated genes, whereas downregulated genes disproportionately mapped to long noncoding RNAs (lncRNAs) in the multisite analysis (Figure 1C).

### Upregulation of diverse inflammatory pathways in EED

GSEA to identify over-represented biological and functional pathways associated with the EED transcriptome demonstrated a disproportionate representation of immune pathways and processes (Figure 1D). The upregulated genes in EED were enriched across diverse functional categories, including humoral immunity, viral response, T-helper 17 (Th17) differentiation, and IL-17, TNF, toll-like receptor, janus kinase/signal transducer and activator of transcription (JAK/STAT), and IL-18 signaling. Digestion, mineral absorption, detoxification, and stress response to metal ions were over-represented among the downregulated genes, suggesting these functions are impaired in EED (Figure 1D) (complete GSEA results in Supplemental Data set 2).

### The Lcn2 gene module of host defense in EED

**FIGURE 2:**
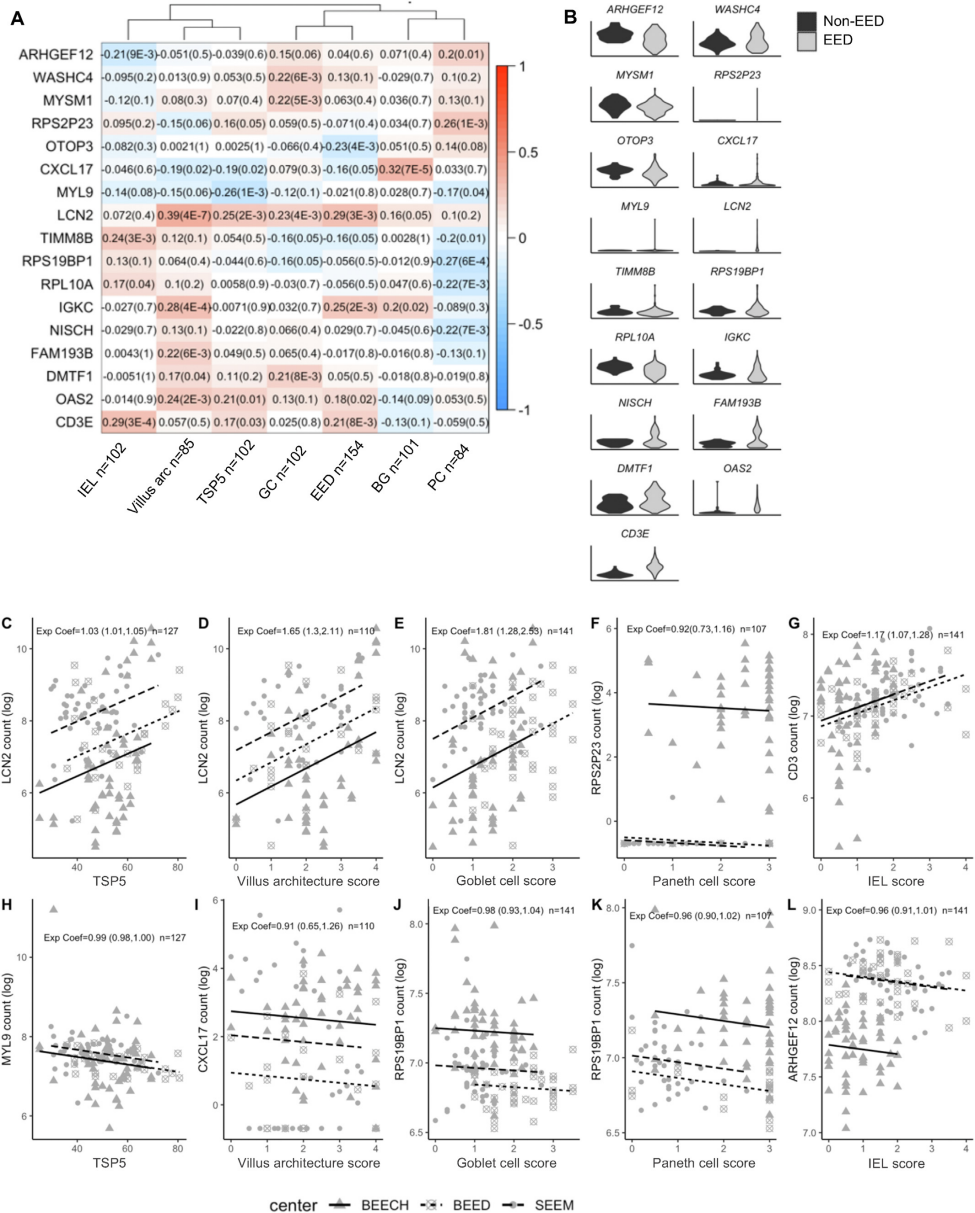
**Gene coexpression modules in EED.** (A) Heatmap of gene modules associated with the EED histologic index score and component histologic scores. Colors correspond to the Pearson correlation coefficient. *P* values adjusted for the number of comparisons using a Bonferroni correction are shown in parentheses. Correlations with an absolute value <0.2 were omitted for simplicity (complete results are in Supplemental Data set 3). (B) Expression of hub genes in EED (gray *n* = 141) and non-EED (black *n* = 39) cohorts. (C–L) Relationship between module hub gene expression and corresponding histologic scores. Coefficients were estimated using mixed-effects linear regression, adjusting for site, and RNA counts are log transformed. Only subjects with histologic scores were included (TSP-5 = 127, villus architecture = 110, goblet cell score = 141, Paneth cell score = 107, and IEL = 141).

**FIGURE 3:**
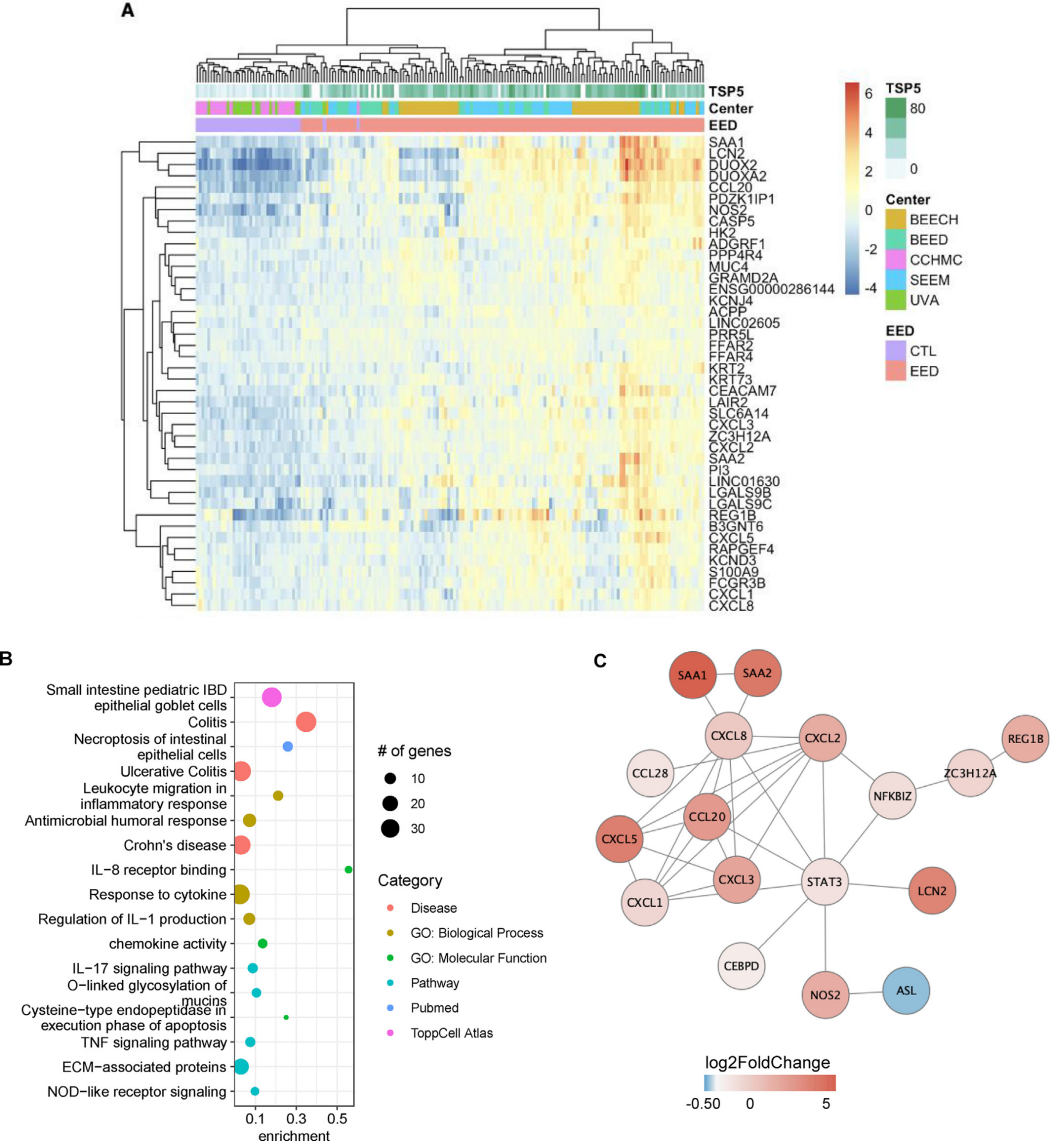
**The*LCN2*module contains coregulated antimicrobial genes.** (A) Heatmap of differentially expressed genes in the *LCN2* module. (B) ToppGene enrichment analysis of the *LCN2* module. Dot size corresponds to the number of genes in the enriched category. Dot color indicates the database source. Enrichment was calculated as [# of genes in query geneset/# of genes in genome]. (C) Network plot of *LCN2* gene module protein–protein interactions. Node color corresponds to LFC values in EED.

We used WGCNA to identify networks of coexpressed genes correlated with EED (Figure 2A, Supplemental Data set 3). Associated modules are referred to by their hub genes, i.e., the gene with the greatest connectivity in the module (Supplemental Data set 4). The expression of hub genes in EED and comparison groups is shown in Figure 2B. The *LCN2* module was the most strongly associated with EED diagnosis (*R* = 0.29, *P*_adj_ *=* 0.0003) and *LCN2* was significantly upregulated in children with EED (LFC = 3.88, *P*_adj_ *=* 3.76E-34) (Figure 2B). *LCN2* encodes a neutrophil gelatinase-associated lipocalin that limits bacterial growth. Differentially expressed genes in the *LCN2* gene module included *DUOX2, DUOX2A, SAA1,* C-C motif chemokine ligand 20 (*CCL20*), and *REG1B* (Figure 3A). Overall, this module was enriched for genes with chemokine activity (*P*_adj_*=*2.9 E-04), necroptosis of intestinal epithelial cells (*P*_adj_*=*2.9E-08), antimicrobial humoral response (*P*_adj_*=*9.9 E-06), and *O*-linked glycosylation of mucins (*P*_adj_=5.1 E-03). The *LCN2* module was also enriched for genes associated with ulcerative colitis (*P*_adj_*=*1.79E-06) and Crohn’s disease (*P*_adj_*=*1.4E-05) (Figure 3B, Supplemental Data set 5A).

Protein–protein interaction analysis of the *LCN2* module revealed a core set of 17 differentially expressed genes that form a high-confidence protein network (Figure 3C). The 17 interacting proteins were significantly upregulated at the transcript level, except for argininosuccinate lyase (*ASL*)*,* which was modestly downregulated (LFC = −0.34, *P*_adj_= 8.27E-6) in EED. The inverse regulation of nitric oxide synthase 2 (*NOS2*) and *ASL* has been observed in gastric cancer [42] and suggests arginine metabolism and nitric oxide production may be dysregulated in EED. Functional enrichment identified defense response as the key function of the Lcn2 protein network (*P*_adj_ = 9.75 × 10^−11^) with chemokine motifs as a top protein domain (*P*_adj_ = 3.48 × 10^−^11) in this network.

### Other EED-associated gene modules

The Ig kappa constant (*IGKC*) module was also associated with EED (*R*=0.25, *P*_adj_=0.002) (Figure 2A) and was enriched for Ig complex genes (*P*_adj_*=* 5.6 E-111) and B cell-mediated immunity (*P*_adj_*=* 3.1 E-61*)* (Supplemental Data set 5B)*.* The CD3 Epsilon Subunit Of the T-Cell Receptor Complex (*CD3E*) module was the third most associated module with EED and was highly enriched for pathways associated with T-cell activation (*P*_adj_*=* 5.7E-42), and antigen presentation including major histocompatibility complex (MHC) binding (*P*_adj_*=*2.8 E-9), guanosine triphosphate (GTP)-ase activity (*P*_adj_*=*1.2 E-07), and transporter associated with antigen presentation (TAP) binding (*P*_adj_*=*1.46E-06) (Supplemental Data set 5C).

In contrast, the otopetrin 3 (*OTOP3*) module was negatively associated with EED (R=−0.23, *P*_adj_*=*0.004) and *OTOP3* expression was decreased in EED (LFC = −0.55, *P*_adj_=1.5 E-5) (Figure 2B). *OTOP3* encodes a proton-selective channel involved in pH sensing and regulation of membrane potential [43] and the *OTOP3* module was enriched for genes related to the brush border assembly (*P*_adj_*=*2.4E-07), microvillus organization (*P*_adj_*=*1.0 E-04), and cellular signatures of mature duodenal enterocytes (*P*_adj_*=*2.2 E-33) (Supplemental Dataset 5D).

### Gene modules associated with EED histopathologic features

We next examined the relationship between the expression of top module hub genes and their associated histopathologic features in the EED cohort. The *LCN2* module was the most highly correlated with EED severity based on TSP5 (*R*=0.25, *P*_adj_=0.002), villus blunting (*R* = 0.39, *P*_adj_ = 4 E-07), and goblet cell paucity (*R*=0.23, *P*_adj_=0.04) (Figure 2A). Expression of *LCN2* positively correlated with each of these features (Figure 2C–H) across EED centers. In contrast, the ribosomal protein S2 pseudogene 23 (*RPS2P23*) module was the most associated with PC depletion (*R* = 0.26, *P*_adj_ = 0.001) (Figure 2A) but *RPS2P23* expression was not (Figure 2F**)**.

IEL infiltration was most highly correlated with the *CD3E* module (*R* = 0.29, *P*_adj_=0.0003). (Figure 2A). The hub gene *CD3E* encodes a subunit of the T-cell receptor, which is expected to be highly expressed by IELs, so this observation adds confidence to WGCNA for identifying biologically relevant gene expression signals associated with specific histologic features. As further validation of this approach, *CD3E* expression and IEL infiltration score were significantly correlated across EED centers (Figure 2G).

**FIGURE 4:**
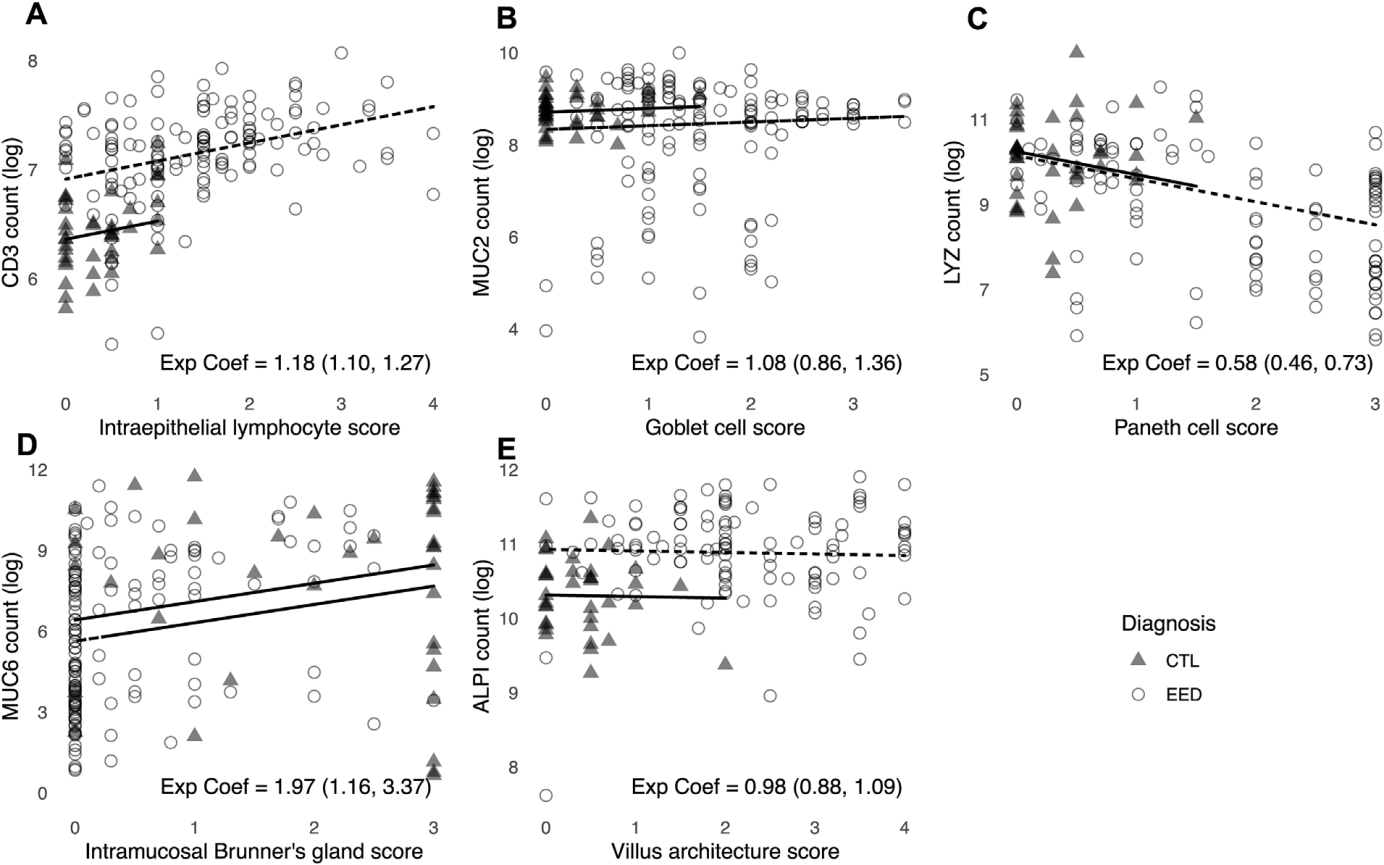
**Expression of genes related to the histologic features of EED**. The relationships between the expression of selected gene markers (*Y*-axis) and their respective EED histopathologic feature (*X*-axis) were evaluated in the EED and comparison cohorts by a linear mixed-effects model. Samples without histologic scores were omitted. RNA counts were log transformed and exponentiated coefficients and 95% CIs are shown on each plot. (A) IEL infiltration score and *CD3E* expression (*n* =180). (B) Goblet cell depletion score and *MUC2* expression (*n* = 180). (C) Paneth cell depletion score and *LYZ* expression (*n* = 180). (D) Brunner’s gland score and *MUC6* expression (*n* = 178). (E) Villus architecture score and *ALPI* expression (*n* = 146).

Correspondingly, for modules with negative correlations with histologic features by WGCNA, expression of hub genes was inversely correlated with worsening EED scores. The myosin light chain 9 (*MYL9*) module was most negatively correlated with EED severity by TSP5 score (*R* = −0.26, *P*_adj_=0.001) (Figure 2A) and *MYL9* expression was negatively correlated with TSP-5 score (Figure 2H**)**. The *MYL9* module was enriched for genes involved in extracellular matrix organization (*P*_adj_*=*6.2E-19) and pediatric small intestine mesenchymal–myocytic cell signatures (*P*_adj_*=*1.2E-124) (Supplemental Data set 5E) suggesting these functions are increasingly depleted as EED becomes more severe. The C-X-C motif chemokine ligand 17 (*CXCL17*) gene module was negatively correlated with villus architecture (R=−0.19, *P*_adj_=0.02) (Figure 2A), and *CXCL17* expression was reduced relative to increased villus damage (Figure 2K). The *CXCL17* module was enriched for genes related to small intestinal epithelial tuft-related microfold cells (*P*_adj_*=*7.1 E-66) and digestion (*P*_adj_*=*3.4 E-03) (Supplemental Data set 5F). Likewise, the *CXCL17* module was most positively correlated with intramucosal Brunner’s glands scores (Figure 2A) which are less dense in EED (Table 2). However, expression of *CXCL17* was not associated with the Brunner’s gland score (data not shown). *MUC6* was also a highly connected gene in the *CXCL17* module and is a known marker of Brunner’s glands, and we found *MUC6* expression to be correlated with Brunner’s gland scores (Figure 4D). The ribosomal protein S19-binding protein 1 (*RPS19BP1*) module was negatively correlated with both goblet (*R* = −0.16, *P*_adj_=0.05) and PC depletion (*R* = −0.27, *P*_adj_=0.0006), and *RPS19BP1* expression was negatively correlated with histologic scores for both features across centers (Figure 2J, K).

### Transcriptional responses are correlated with histologic features of EED

To further explore the relationship between small intestinal gene expression and histopathology we examined the expression of top cellular markers associated with EED compared with the comparison cohorts (Figure 4). As in the EED cohort (Figure 2G), *CD3E* expression was associated with IEL infiltration scores across both study groups (Figure 4A). We found no association between *MUC2* expression and goblet cell depletion (Figure 4B**),** but expression of lysozyme (*LYZ)*, a canonical PC maker [44], was significantly correlated with PC depletion (Figure 4C). As expected, expression of *MUC6*, a marker for duodenal Brunner’s glands [45] (reduced in EED), correlated with increased Brunner’s glands (Figure 4D). Unexpectedly, expression of alkaline phosphatase (*ALPI*), a marker of differentiated enterocytes [46], was positively correlated with villus architecture scores, suggesting a transcriptional shift toward differentiation and lipid absorption in response to villus injury.

### Transcriptional responses correlate with protein expression in EED

**FIGURE 5:**
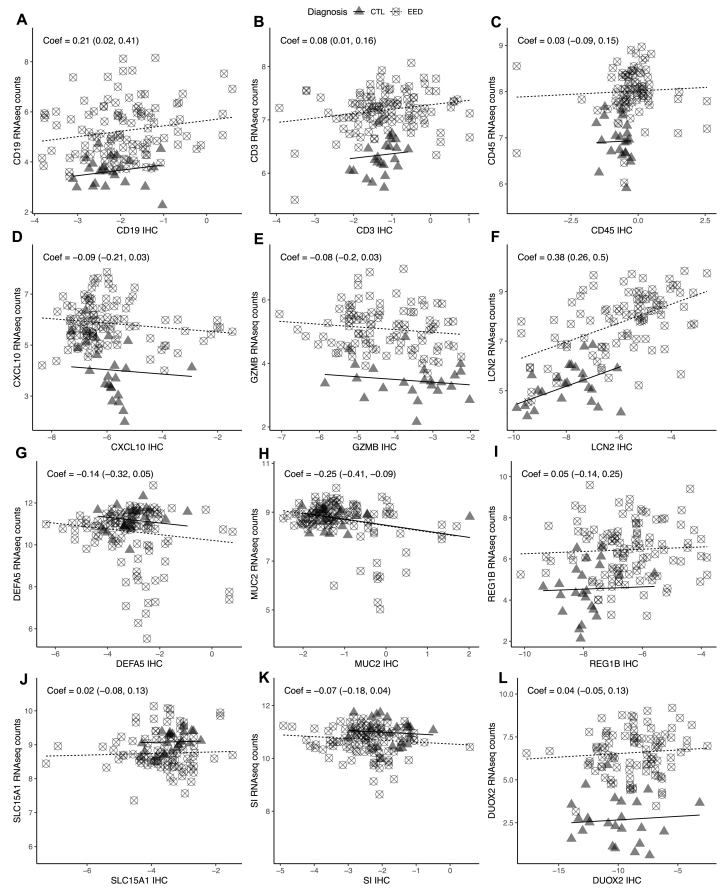
**Relationship between quantitative immunohistochemical markers of EED and small intestinal gene expression.** The relationship between the transcript abundances (*X*-axis) and quantitative immunohistochemical measure of protein (*Y*-axis) in paired biopsies was evaluated using a linear mixed-effects model. Symbols correspond to the study group. Transcript abundances and IHC measures are log transformed and coefficients and 95% CIs are shown on each plot. Only samples with IHC values are included. (A) CD19 (*n* = 132); (B) CD3 (*n* = 132); (C) CD45 (*n* = 108); (D) CXCL10 (*n* = 131); (E) GZMB (*n* = 118); (F) LCN2 (*n* = 119); (G) DEFA5 (*n* = 130); (H) MUC2 (*n* = 132); (I) REG1B (*n* = 131); (J) SLC15A1 (*n* = 111); (K) SI (*n* = 132); and (L) DUOX2 (*n* = 118).

To investigate the relationship between gene regulation and protein expression in the small intestine we correlated analysis of proteins measured by IHC and gene transcripts in the EED and comparison groups (Figure 5). Three of the gene markers (*CD19*, *CD3*, and *LCN2*) were positively associated and one was negatively associated (*MUC2*) with their corresponding proteins as measured by IHC (Figure 5 and Supplemental Table 3)

## Discussion

This multicenter transcriptomic analysis of duodenal tissue from children with EED offers insight into tissue responses that underlie this disorder, many of which resemble those in other inflammatory intestinal disorders, including celiac disease, Crohn's disease, and intestinal infections. However, our data also raise questions. For example, some upregulated genes (e.g., *CCL3, CCL4, RHOH, EGR2, CXCL1, CXCL2,* *TNF*, *IFNG*, *LCN2* (Figure 1, Supplemental Dataset 1)) infer a presence of and role for neutrophils, but these are not present in EED biopsies [1,9].

*LCN2*, a neutrophil gelatinase-associated lipocalin, warrants additional comment. Lcn2 was initially identified as a protein secreted by neutrophils [47], however epithelial cells are also capable of secreting large amounts of this gelatinase [48]. In mice, *LCN2* modulation of microbiota is essential for maintaining intestinal homeostasis during chronic inflammation resulting from loss of IL-10 [49]*.* *LCN2* may fulfill a similar role in EED as it was coordinately induced with other antimicrobial response genes (e.g., *S100A9, NOS2, SAA1, SAA2*) and proinflammatory pathways (TNF signaling, IL-17 signaling, IL-8 receptor binding) (Figure 3B, supplemental dataset 5A). The Lcn2 protein interaction network centered on Stat3 (Figure 3C) which induces *LCN2* expression in response to inflammatory cytokines [50]. Lcn2 also opposes inflammatory processes via modulation of NFκB-Stat3 activation [51]. The identification of two anti-inflammatory regulators NFκB inhibitor zeta (Nfkbiz) and the zinc finger CCCH-type containing 12A endoribonuclease (Zc3h12a) in the Lcn2 EED protein network (Figure 3C) raises the possibility that coordinate induction of both pro-and anti- inflammatory regulators maintain intestinal homeostasis in EED. Investigating the role of Lcn2 in this interplay is worthy of further pursuit.

*REG1B*, a marker of tissue response to injury, was among the coregulated genes in the *LCN2* module. Fecal Reg1b is a biomarker of intestinal injury and repair and a predictor of future childhood stunting [52]. Reg1b was identified as a key duodenal protein related to specific microbial taxa in the BEED cohort [53], and, in a recent study of metabolic syndrome, *REG1B* was profoundly induced by the butyrogenic *Anaerobutyricum soehngenii* [54] Stimulation of Reg1 signaling might hasten epithelial repair and be a target for treating EED, for example via prebiotic or probiotic interventions.

The IL-17A-IL-22 axis also emerged as a key transcriptionally regulated element of mucosal responses in EED. Th17 cells mount defenses against bacterial and fungal pathogens at mucosal surfaces, in particular the small intestine. We found, as in celiac disease, strong upregulation of IL-17 signaling and Th17 cell differentiation in EED (Figure 1E) including induction of *IL21, IL6, IL17A IL17F, IL22, IFNG, IL21R,* and *IL2RA* (Supplemental Dataset 1). Secretion of these cytokines and expression of their receptors in the small intestine induces secretion of molecules, such as Lcn2, which could protect the mucosal barrier and prevents pathogen dissemination [55,56]. The *LCN2* module is enriched for downstream targets of IL-17 signaling (Figure 3B) including *LCN2*, *S100A9, CCL20,* and *CXCL1,2,3,5,8*- defined previously.

In addition to innate protective effects, Th17 cells are implicated in celiac disease pathogenesis, where gluten and bacteria trigger IL-17A responses in the intestinal mucosa [57,58], and are also associated with villus atrophy [59]. Th17 differentiation highly depends on commensal microbes and might be induced by microbial alterations in EED. There is precedent for the intestinal microbiota eliciting protective Th17 differentiation [60]. As such IL-17 signaling may be a target for microbiota-directed interventions in EED, but neutralization of IL-17 with monoclonal antibodies in inflammatory bowel disease paradoxically exacerbated symptoms [61]. We suggest IL-17 predicts villous atrophy in EED and should be carefully evaluated further as a therapeutic target.

We defined the EED transcriptome in relation to a North American comparison group that is unexposed to the environmental triggers of EED. A chart review was performed to exclude children with current or previous gastrointestinal diagnosis and a pathologic review confirmed histologically normal tissue. Nonetheless, the indication for endoscopy suggests that unaccounted gastrointestinal disturbances may exist. North American children also have distinct diets, microbiota, infectious disease burden, and genetics, among other factors, that likely impact the intestinal transcriptome. As such, unaffected children from the same populations as the EED cohorts would be a superior comparison group however we viewed this as an inherent limitation as performing endoscopies on healthy children is not ethically acceptable. Nonetheless, there is high confidence based on the diagnosis, pathology evaluation, and geographic location that, at the bare minimum, the comparison group is composed of children without EED. The comparison group also included a broader age range to enroll sufficient children during the study period. Although age differed significantly between the EED and comparison group cohorts, age was not a significant covariate according to our model selection, suggesting that the effect of age on gene expression is minor. We also found that age did not significantly affect gene expression in the comparison group samples alone (Supplemental Figure 3F).

The intestinal transcriptome of EED presents gene profiles associated with response to pathogens (Figure 1D). This is consistent with findings in a companion manuscript that children with EED harbored a high fecal enteric pathogen burden in the absence of diarrhea symptoms before biopsy [62]. Assessing relationships between enteropathogen burden and gut inflammation in EED is an important area for future investigation.

We also acknowledge inherent technical limitations from differences in library preparation and RNA-seq, and despite the use of quality controls for each stage of sample preparation, potential biases inherent in experimental design might have been introduced in a center-specific manner. We attempted to reduce differences by using uniform data processing, read alignment, transcript quantification, normalization, and differential expression analysis, and attempted to mitigate potential confounding variables in our analytical model selection.

The strength of our work includes the integration of multiple prospective studies and the use of endoscopy to collect tissue which together offer an unprecedented insight into the small intestinal tissue transcriptome in EED. A further strength is the integration of data across 3 geographic areas which adds confidence that these results have relevance to children with EED across geographic locations. Despite differences in environmental exposures and genetic backgrounds of the cohorts, we identified core transcriptional elements of EED that offer high priority targets for future interventional studies. Each child that was biopsied in the 3 EED cohorts had aberrant histology and transcriptomic evidence of enteropathy. Based on its high prevalence, and strong associations with malnutrition, growth faltering, and oral vaccine failure, EED presents an immediate opportunity for novel interventions to prevent or ameliorate this disorder, and transcriptional analysis suggests target pathways.

## Acknowledgments

We thank the patients and their families for their generous participation in this study, the CCHMC sequencing core (Core ID P30 DK078392-16) for performing the RNA-seq of the CCHMC samples, and the UVA Bioinformatics Core for assistance with the WCGNA analysis (University of Virginia Strategic Investment Fund #162 TransUniversity Microbiome Initiative). We thank Louise Warren for guidance with manuscript proofing, formatting, and submission.

## Author contributions

The authors’ responsibilities were as follows − CM, PK, WAP, LAD, TA, MM, SAA,SRM: designed research; CM, PK, WAP, SD, JI, LAD, TA, MM, SAA, SRM: conducted research; PIT: data management; CM, LAD, DC: analyzed data; CM, PIT, LAD: data interpretation; CM: wrote the paper and has primary responsibility for final content; and all authors read and approved the final manuscript.

## Conflict of interest

The authors report no conflicts of interest.

## Funding

The EEDBI Consortium was funded by the following grants: Bill and Melinda Gates Foundation OPP1152812,OPP1066118, OPP1136759, OPP1138727, and OPP1144149, and Advanced Imaging and Tissue Analysis Core of the Washington University Digestive Diseases Research Core Center P30DK052574, and Gene Expression Core of the Cincinnati Children’s Hospital Medical Center Digestive Diseases Research Core Center P30DK078392. WAP was funded by R01AI43596. CM was funded by R01AI148518.

## Data availability

Data described in the manuscript, code book, and analytic code will be made available upon request to the corresponding author pending application and approval.

## Figures and Tables

**Table:** 

Surname	Name	Full Affiliation	Email
Ahmed	Kumail	Department of Paediatrics and Child Health, Aga Khan University, Karachi, Pakistan	kumail.ahmed@aku.edu
Ahmed	Sheraz	Department of Paediatrics and Child Health, Aga Khan University, Karachi, Pakistan	sheraz.ahmed@aku.edu
Alam	Md Ashraful	Nutrition Research Division, International Centre for Diarrhoeal Disease Research, Bangladesh, Dhaka, Bangladesh	mashraful@icddrb.org
Auble	David	Department of Biochemistry and Molecular Genetics, University of Virginia School of Medicine, Charlottesville, Virginia, USA	dta4n@virginia.edu
Begum	SM Khodeza Nahar	Department of Pathology, Bangladesh Specialized Hospital, Dhaka, Bangladesh	khodeza33@hotmail.com
Besa	Ellen	Tropical Gastroenterology & Nutrition Group, University of Zambia School of Medicine, Lusaka, Zambia	ellen@tropgan.net
Chama	Mubanga	Tropical Gastroenterology & Nutrition Group, University of Zambia School of Medicine, Lusaka, Zambia	mubangachama@yahoo.co.uk
Denno	Donna M	Department of Pediatrics, University of Washington School of Medicine, Seattle, WA, USA	ddenno@uw.edu
Fahim	Shah Mohammad	Nutrition Research Division, International Centre for Diarrhoeal Disease Research, Bangladesh, Dhaka, Bangladesh	mohammad.fahim@icddrb.org
Gazi	Md Amran	Nutrition Research Division, International Centre for Diarrhoeal Disease Research, Bangladesh, Dhaka, Bangladesh	amran.gazi@icddrb.org
Haberman	Yael	Division of Pediatric Gastroenterology, Hepatology, and Nutrition, Cincinnati Children’s Hospital Medical Center, Cincinnati, OH, USA	Yael.Haberman@cchmc.org
Haque	Rashidul	Infectious Disease Division, International Centre for Diarrhoeal Disease Research, Bangladesh, Dhaka, Bangladesh	rhaque@icddrb.org
Hasan	Md Mehedi	Nutrition Research Division, International Centre for Diarrhoeal Disease Research, Bangladesh, Dhaka, Bangladesh	md.hasan@icddrb.org
Hossain	Md Shabab	Nutrition Research Division, International Centre for Diarrhoeal Disease Research, Bangladesh, Dhaka, Bangladesh	dr.shabab@icddrb.org
Hotwani	Aneeta	Department of Paediatrics and Child Health, Aga Khan University, Karachi, Pakistan	aneeta.hotwani@aku.edu
Iqbal	Najeeha Talat	Department of Paediatrics and Child Health, Aga Khan University, Karachi, Pakistan	najeeha.iqbal@aku.edu
Jan	Ning-Jiun	Department of Medicine, University of Virginia School of Medicine, Charlottesville, Virginia, USA	gtv7kc@virginia.edu
Kabir	Furqan	Department of Paediatrics and Child Health, Aga Khan University, Karachi, Pakistan	furqan.kabir@aku.edu
Kumar	Pankaj	Department of Biochemistry and Molecular Genetics, University of Virginia School of Medicine, Charlottesville, Virginia, USA	pk7z@virginia.edu
Liu	Ta-Chiang	Department of Pathology and Immunology, Washington University School of Medicine, St. Louis, MO, USA	ta-chiang.liu@wustl.edu
Mann	Barbara J.	Department of Medicine, University of Virginia School of Medicine, Charlottesville, Virginia, USA	bjm2r@virginia.edu
Mazumder	Ramendra Nath	Nutrition Research Division, International Centre for Diarrhoeal Disease Research, Bangladesh, Dhaka, Bangladesh	ramen@icddrb.org
Mohammad	Anwaruddin	Department of Biochemistry and Molecular Genetics, University of Virginia School of Medicine, Charlottesville, Virginia, USA	xdh9bx@virginia.edu
Moskaluk	Christopher A	Department of Pathology, University of Virginia School of Medicine, Charlottesville, VA, USA	cam5p@virginia.edu
Nayak	Uma	Department of Public Health Sciences, University of Virginia School of Medicine, Charlottesville, Virginia, USA	un8x@virginia.edu
Ndao	Malick I	Department of Pediatrics, Washington University School of Medicine, St. Louis, MO, USA	ndao_m@wustl.edu
Ragahavan	Shyam S	Department of Pathology, University of Virginia School of Medicine, Charlottesville, VA, USA	SR7WQ@hscmail.mcc.virginia.edu
Rahman	Masudur	Department of Gastroenterology, Sheikh Russel National Gastroliver Institute and Hospital, Dhaka, Bangladesh	drmasud47@yahoo.com
Rehman	Najeeb	Department of Paediatrics and Child Health, Aga Khan University, Karachi, Pakistan	najeeb.rahman@aku.edu
Sadiq	Kamran	Department of Paediatrics and Child Health, Aga Khan University, Karachi, Pakistan	kamran.sadiq@aku.edu
Sarker	Shafiqul Alam	Nutrition Research Division, International Centre for Diarrhoeal Disease Research, Bangladesh, Dhaka, Bangladesh	sasarker@icddrb.org
Shaikh	Nurmohammad	Department of Pediatrics, Washington University School of Medicine, St. Louis, MO, USA	shaikh_n@wustl.edu
Sullivan	Peter B	Department of Paediatrics, Children's Hospital, University of Oxford, Oxford, UK	peter.sullivan@paediatrics.ox.ac.uk
Tearney	Guillermo J	Department of Pathology, Harvard Medical School, Boston, MA, USA	gtearney@partners.org
Umrani	Fayaz	Department of Paediatrics and Child Health, Aga Khan University, Karachi, Pakistan	fayaz.umrani@aku.edu
Yilmaz	Omer H	Department of Pathology, Massachusetts General Hospital, Boston, MA, USA	oyilmaz@mgh.harvard.edu
Zyambo	Kanekwa	Tropical Gastroenterology & Nutrition Group, University of Zambia School of Medicine, Lusaka, Zambia	kanekwa@tropgan.net
