# Supplementary material for: Duodenal transcriptomics demonstrates signatures of tissue inflammation and immune cell infiltration in children with environmental enteric dysfunction across global centers
Source: Am J Clin Nutr. 2024 Sep 17;120(Suppl 1):S51–64. doi: 10.1016/j.ajcnut.2024.02.023 (PMC11562032; doi:10.1016/j.ajcnut.2024.02.023)
Supplement: Multimedia component 2 [file mmc2.zip › ajcnut_465_CHELSE~2_mmc2.DOC]

**Transcriptomics supplemental appendix**

1. **Supplemental Methods**
   1. Ethical approval
   2. Study Design: enrollment criteria, intervention details and study dates
   3. Duodenal biopsy sample collection, RNA extraction and sequencing
   4. Intersite Differential Expression Analysis of EED
   5. Histopathologic evaluation of small intestinal biopsies
   6. Variables
2. **Supplemental Results**
   1. Integration of RNA-seq data across centers
   2. Differential Expression Model Selection
   3. Sensitivity analysis
   4. Intersite Variability of EED
3. **Supplemental Tables**

Supplemental Table 1: RNA-Sequencing Methods Across Centers

Supplemental Table 2: Histologic Scoring Criteria for TSP-5

Supplemental Table 3: Protein and Transcript Correlations in Duodenal Tissue

1. **Supplemental Figures**

Supplemental Figure 1: Flow chart of Enrollment by Study Group.

Supplemental Figure 2: Sequencing Library Characteristics

Supplemental Figure 3: Effect of Covariates on Gene Expression

Supplemental Figure 4. Sensitivity Analysis of Differential Expression Supplemental Figure 5: Intersite Variability of EED

**Supplemental Methods**

**Ethical Approval**

Ethical approval was obtained from AKU Ethics Review Committee (ERC) (3836-Ped-ERC-15), icddr,b ERC (PR-16007), University of Zambia Biomedical Research Ethics Committee (006-02-16) and the National Health Research Authority (MH/101/23/10/1), UVA Institutional Review Board (19466), CCHMC Institutional Review Board (2016-0387). Exemption was received from the University of Washington Institutional Review Board (IRB) (STUDY00013442) and the Washington University IRB (201801207).

**Study Design: enrollment criteria, intervention details and study dates**

An overview of study enrollment by site is in **Supplemental Figure 1**.

*BEECH*

BEECH enrollment criteria were children 1-18 months of age with wasting or stunting (WAZ, LAZ, or WLZ <-2). Exclusion criteria for BEECH were caregiver unwilling for child to undergo Human Immunodeficiency Virus (HIV) test and receive HIV care (if relevant), and if participating in another research study. The BEECH intervention consisted of counseling on nutrition, water/sanitation/hygiene, and home care of child illness, with breastfeeding support and education. High energy protein supplement (corn-soya blend), 14 Eggs and 14 sachets of micronutrient powder were provided to children 6 months and older every two weeks. Children with complicated SAM received hospital management and those with uncomplicated SAM were managed with ready-to-use therapeutic feeds on an outpatient basis per national protocols. Children with LAZ or WLZ consistently <-2 after 3-4 months of nutritional supplementation were eligible for endoscopy. BEECH collected 2 biopsies for RNA-sequencing from the first 30 children biopsied without selection. The recruitment period for BEECH occurred between October 1 2016 – May 31 2018, and biopsies for RNA-seq were collected between February 2, 2017 - June 7, 2019.

*BEED*

BEED enrollment criteria were children aged 12-18 months with LAZ <= -1. Exclusion criteria for BEED were severe acute malnutrition, history of persistent diarrhea, known allergy to eggs or milk or milk intolerance. BEED intervention consisted of directly observed on-site feeding of boiled egg and 150 ml whole milk at a study nutrition center 6 days/week for 90 days, in conjunction with anti-helminthic treatment per national guideline, micronutrient sprinkles (one sachet daily for two months), and nutritional counseling for caregivers. Children that did not attain an improvement in LAZ during the 90 day intervention were eligible for endoscopy. RNA-seq was conducted on the first 41 children to undergo biopsy without selection. The recruitment period for BEED was between July 17, 2016 - May 31, 2019 and biopsies for RNA-seq were collected between November 2, 2016 - August 26, 2019.

*SEEM*

SEEM enrollment criteria were 3-6 months old and WLZ<-2. Exclusion criteria for SEEM were WLZ>0 and LAZ not <-1 on two consecutive visits. The SEEM intervention consisted of nutritional counseling for caregivers and nutritional supplementation starting at 9 months of age. For children with AchaMuM - a ready-to-use supplementary food - 1 sachet daily for 2 months for WLZ<-2 but >-3. For WLZ<-3, weight-based supply was provided. Children that did not show improvement in height and weight compared to preceding weight and height were eligible for endoscopy. RNA-seq was performed on the first 49 children biopsied (1).The recruitment period for SEEM was between July 17, 2016 - May 31, 2019 and biopsies for RNA-seq were collected between January 11, 2017 - October 10, 2018.

*CCHMC*

The CCHMC comparison group was enrolled at CCHMC with inclusion criteria of <12 years of years of age and presenting for endoscopic intestinal biopsy for diagnostic purposes. Exclusion criteria were 1. Recent antibiotic treatment (≤4 weeks prior to endoscopy, known coagulopathy, thrombocytopenia, or bleeding disorder, severe chronic medical condition unrelated to IBD, known connective tissue disorder (e.g. Marfan syndrome or confirmed Ehlers Danlos Type IV syndrome) or eosinophilic gastrointestinal disease (eosinophilic esophagitis), known immunodeficiency, or, at the discretion of the investigator, any other condition that would make taking additional biopsies unsafe for the patient. Children who underwent endoscopy and had a diagnostic histology consistent with esophagogastrointestinal disease were excluded from the RNA-seq analysis (n=17). The nondiagnostic group, comprising those without medical diagnoses or diagnostic histology consistent with esophagogastrointestinal disease (n=23) was included in the RNA-seq comparison group. Enrollment, endoscopies, and biopsies took place between March 24, 2017 - March 5, 2019.

*UVA*

The UVA comparison group was enrolled with inclusion criteria of 1-18 years of age presenting for endoscopic intestinal biopsy for diagnostic purposes. The exclusion criteria were parent or guardian unwilling to consent. Children who underwent endoscopy with diagnostic histology consistent with esophagogastrointestinal disease (such as eosinophilic esophagitis, gastritis, celiac and inflammatory bowel disease) were excluded from the RNA-seq analysis (n=74). The nondiagnostic group, comprising those without medical diagnoses or diagnostic histology consistent with esophagogastrointestinal disease (n=16) was included in the RNA-seq comparison group. Enrollment, endoscopies, and biopsies took place between June 7, 2017 - September 13, 2019.

**Duodenal biopsy sample collection, RNA extraction and sequencing**

A summary of methods by site is presented in **Supplemental Table 1**.

*BEECH*

Detailed methods have been described (2). Briefly, RNA-sequencing was performed on biopsies from the first 30 consecutive children to be declared to have non-responsive stunting, without selection. Two small bowel biopsies were immediately snap-frozen in liquid nitrogen before storage at −80°C. RNA was extracted using TRIzol (Invitrogen) followed by silica column purification (RNeasy Mini Kit; QIAGEN) and quantified using a NanoDrop spectrophotometer (ND-2000C; Thermo Fisher Scientific) before transport to the Beijing Genomics Institute. RNA quality control was performed using an Agilent 2100 Bioanalyzer and ABI StepOnePlus Real-Time PCR System. For RNA-seq preparation, total RNA was treated with DNase I followed by mRNA enrichment using oligo-deoxythymine-labelled beads and ligation of sequencing adaptors to the enriched mRNA fragments. RNA-seq was performed using an Illumina HiSeq 2000 Sequencing System with 50 bp paired-end reads.

*BEED and UVA*

One duodenal biopsy obtained during diagnostic esophagogastroduodenoscopy was immediately placed in Allprotect (Qiagen, Valencia, CA) and stored at -80^o^C until RNA extraction using the Qiagen AllPrep RNA/DNA Mini Kit (Qiagen, USA). 1 μg of RNA per sample was used as input material for the mRNA Library preparations. Sequencing libraries were generated using NEBNext UltraTM RNALibrary Prep Kit for Illumina (NEB, USA) and the NEBNext Multiplex Oligos for Illumina (NEB, USA) following the manufacturer’s recommendations. mRNA was purified from total RNA using poly-T oligo-attached magnetic beads. cDNA fragments of preferentially 150~200 bp in length, the library fragments were purified with AMPure XP system (Beckman Coulter, Beverly, USA). PCR products were purified (AMPure XP system) and library quality was assessed on the Agilent Bioanalyzer 2100 system. Clustering of index-coded samples was performed on a cBot Cluster Generation System using PE Cluster Kit cBot-HS (Illumina) according to the manufacturer’s instructions. After cluster generation, the library preparations were sequenced on an Illumina MiSeq and 150 bp paired-end reads were generated (Novogene, USA).

*SEEM and CCHMC*

Detailed methods are available in the original study descriptions (1). Total RNA was isolated from one duodenal biopsy obtained during esophagogastroduodenoscopy using the Qiagen AllPrep RNA/DNA Mini Kit (Qiagen, Valencia, CA). mRNA was enriched by PolyA-RNA selection. mRNA fragmentation, cDNA synthesis, adaptor ligation, TruSeq RNA sample library preparation (Illumina, San Diego, CA), and paired-end 75bp sequencing were performed by the CCHMC Digestive Health Center core facility on the Illumina HiSeq 2500, NextSeq 500, and NovaSeq 6000 platforms.

**Intersite Differential Expression Analysis of EED**

Genes differentially expressed (DE) between EED and comparison groups were selected using a Bonferroni adjusted *P* value (*P*_adj_) < 0.05 using the R package DESeq2 (3). Sex and percent intronic bases were included in the differential expression model based on multivariate regression performed for cross-site comparison (4) and LFC shrinkage was applied to differential expression gene sets for visualizing and ranking of genes (5). Differential expression was performed in R version 4.2.1. Biotypes are from biomart annotations (6), the non-coding category includes all ncRNA including polymorphic, transcribed, processed and unprocessed pseudogenes. Functional profiling of site-specific genes was done using g:Profiler for GO terms and pathways (7), the two most significant driver gene ontology (GO) terms for each category are shown as well as the two most significant biologic pathways from KEGG and/or Reactome. Site-specific downregulated genes from SEEM did not have any significant GO or pathway enrichment, but did have significant enrichment for known transcription factor binding sites which are shown.

**Histopathologic evaluation of small intestinal biopsies**

The histology scoring system and procedures are described in a companion paper in this series (8). Briefly, images were semi-quantitatively scored across 8 histologic parameters by two or three gastrointestinal pathologists. The total score percent-5 (TSP-5) was calculated from the five most informative histology parameters for differentiating EED from a reference group of American children: villus architecture (VA), intraepithelial lymphocytes (IEL), goblet cells (GC), Paneth cells (PC), and intramucosal Brunner’s glands (BG). Higher scores reflect increasing  histopathologic severity assessed as increased deviation from the reference group such that higher BG and IEL scores reflect increased density in EED, while higher GC and PC scores reflect lower density in EED, and higher villus architecture scores reflect the increased villus blunting of EED. The scoring criteria and parameters are detailed in Supplemental Table 2. The scoring criteria and parameters are detailed in Supplemental Table 2. If two or more of the five parameters were not scorable because of technical issues, the TSP-5 was considered nonscorable. Scoring criteria and parameters are summarized in **Supplemental Table 2**.

**Variables**

The primary outcome of EED which was considered as a dichotomous variable for the transcriptomic analysis. The diagnostic criteria for EED were growth failure at time of enrollment as defined by LAZ, WAZ, or WLZ, as well as insufficient growth after completion of the study nutritional intervention. Growth was assessed by study personnel using standard methods (9). EED histopathologic scores and quantitative immunohistochemistry values were assessed uniformly in the EED and comparison groups by study pathologists. IHC values and histologic scores were analyzed as continuous variables in relation to co-expressed gene modules in the WGCNA analysis.

Potential confounders included age at time of endoscopy considered as a continuous variable, sex considered as a dichotomous variable, study center considered as a categorical variable, and the percentage of intronic bases in sequencing libraries considered as a continuous variable. Potential effect modifiers identified and controlled for in the differential expression analysis included sex (dichotomous), center (categorical), and percentage of intronic bases (continuous).

**Supplemental Results**

**Integration of RNA-seq data across centers**

This analysis was conducted on RNA-sequencing data generated from biopsies collected by 3 studies at 5 sites listed in Supplemental Table 1 and outlined in **Supplemental Figure 1**. RNA was extracted and libraries were prepared and sequenced at three centers (BEED and UVA, SEEM and CCHMC, and BEECH). All raw sequencing FASTQ files were processed using the same pipeline to reduce center-specific effects. BEECH samples had a notably greater number of total reads, but a greater proportion of intronic and intergenic bases relative to samples from the other two sequencing centers (**Supplemental Figure 2**).

The effect of covariates such as study site, sequencing library characteristics, sex and age were analyzed as part of the data integration strategy (**Supplemental Figure 3**). A principal components analysis (PCA) demonstrated that BEECH samples clustered distinctly from the other centers (Supplemental Figure 3A). This variation is likely caused by technical differences in library preparation and sequencing, including the greater depth but shorter read lengths from BEECH (50 bp versus 75 bp in SEEM/CCHMC and 150bp in BEED/UVA). BEED-UVA and SEEM-CCHMC utilized a paired design whereby RNA extraction, library preparation and sequencing were performed in parallel. Comparison of these centers by PCA without BEECH samples shows that EED drove the variation in principal component 1 (PC1) though center effects are also apparent as the paired EED and comparison group centers were clustered relative to each other (Supplemental Figure 3B). As BEECH samples were extracted, prepared, and sequenced without a North American comparison group, we were unable to assess if similar variance was present in a paired comparison group, thus the relative contribution of technical and biologic sources of variation is difficult to ascertain.

**Differential Expression Model Selection**

We used sageseqr to select an optimal differential expression model based on multivariate regression incorporating relevant covariates. The optimal differential expression model include sex, center, and percent intronic bases as covariates. PCA plots were used to visualize the impact of these covariates on gene expression across centers and samples. The percentage of intronic bases in each library correlated with variation in principal component 1 indicating that inclusion of this covariate in the differential expression model may adjust for a technical effect of library preparation or sequencing (Supplemental Figure 3C). The effect of sex was most apparent in the top 100 most variable genes in principal component 2 (Supplemental Figure 3D). The loadings for this principal component included XIST, the X-inactive specific transcript non-coding RNA on the X chromosome that acts as a major effector of the X-inactivation process. The effect of sex is diminished when the 500 most variable genes are considered, in which case center and EED status drive variation in PC1 and PC2 respectively (Supplemental Figure 3D). The top positive loading gene for PC2 was XIST, the X-inactive specific transcript non-coding RNA on the X chromosome that acts as a major effector of the X-inactivation process, while the top negative loadings were driven by genes on the Y chromosome (Supplemental Figure 3E).

As the comparison group was significantly older than the EED group (Table 1) we sought to test the impact of age on duodenal gene expression. We analyzed differential expression as a function of age in the comparison group alone which had an age range from 2.25-17.6 years old. Six genes were significantly differentially regulated based on age in the comparison cohort (Supplemental Figure 3F).

**Sensitivity analysis**

To probe how the variation in the BEECH samples affects the multi-center differential expression analysis we conducted a sensitivity analysis comparing differential expression in EED with and without participant samples from the BEECH center (**Supplemental Figure 4)**. Both comparisons generated significant differential gene expression with 3172 differentially expressed genes with BEECH included and 2267 differentially expressed genes with BEECH excluded (FDR < 0.05 and abs log2 fold change > 1). There was a high degree of overlap in differentially expressed genes (54.2%, 1912 genes) (Supplemental Figure 4A). In addition, the overall pattern of differential expression was highly similar with and without the BEECH samples (Supplemental Figure 4 B,C). Based on these results we concluded that inclusion of samples from the BEECH study improved the sensitivity of the differential expression analysis, and these samples were included throughout.

**Intersite variability of EED**

To further probe for site specific differences in the EED transcriptome, we performed site-specific differential expression analyses of each EED center relative to the pooled North American comparison group (**Supplemental Figure 5**). BEECH had the greatest number of significantly differentially expressed genes, with many more upregulated genes compared to other sites (Supplemental Figure 5A). This is likely due in part to the higher sequencing coverage and shorter read length (Supplemental Figure 2A). However, the higher proportion of intergenic (Supplemental Figure 2D) and intronic (Supplemental Figure 2E) may also be indicative of incomplete DNA depletion prior to sequencing. This is further supported by the high proportion of non-coding transcripts among the upregulated genes in BEECH (Supplemental Figure 5B), which an overlap analysis found to be largely unique to the BEECH site (Supplemental Figure 5C). Functional profiling of site-specific differentially expressed genes revealed potential unique aspects of EED biology, including a significant enrichment of B-cell and antibody related terms among upregulated genes in SEEM (Immunoglobulin complex, FC-gamma receptor (FCGR) activation and CD22 mediated B cell receptor (BCR) regulation) (Supplemental Figure 5D). Other pathway enrichment specific to individual sites were DNA damage response genes in BEED and increased olfactory signaling in BEECH (Supplemental Figure 5D). Of note, there was no significant enrichment of GO terms or pathways in the SEEM site-specific downregulated geneset (Supplemental Figure 5D). As we were unable to fully differentiate biologic from technical effects, we elected not to explore site-specific differences further.

|  | **BEECH** | **BEED/UVA** | **SEEM/CCHMC** |
| --- | --- | --- | --- |
| **Acquisition** | 2 biopsies snap-frozen in liquid nitrogen | 1 biopsy stored in Allprotect | 1 biopsy stored in RNAlater |
| **RNA extraction** | TRIzol + RNeasy Mini Kit | Qiagen AllPrep RNA/DNA Kit | Qiagen AllPrep RNA/DNA Kit |
| **mRNA enrichment** | oligo(thymine) DNA magnetic beads | oligo(thymine) DNA magnetic beads | oligo(thymine) DNA magnetic beads |
| **Library construction** |  | NEBNext Ultra RNALibrary Prep Kit for Illumina | TruSeq RNA sample library preparation |
| **Sequencing** | 50 bp paired-end, Illumina HiSeq 2000 | 150bp paired-end, Illumina MiSeq | 75bp paired-end ,Illumina HiSeq 2500, NextSeq 500 NovaSeq 6000 |

**Supplemental Table 1: Summary of RNA-sequencing Methods Across Centers**

# **Supplemental Table 2: Histologic Scoring Criteria for TSP-5**

| **Feature** | **Scoring** |
| --- | --- |
| **Intraepithelial lymphocytes (IEL)** | **0:**  lymphocytes/epithelial ratio < = 20% in any area |
|  | **1:** Lymphocyte/epithelial ratio > 20%, but ≤ 50%, in < = 50% of mucosa |
|  | **2:** Lymphocyte/epithelial ratio > 20%, but ≤ 50%, in > 50% of mucosa |
|  | **3:** Lymphocyte/epithelial ratio > 50% in ≤ 50% of mucosa |
|  | **4:** Lymphocyte/epithelial ratio > 50% in > 50% of mucosa |
| **Villus architecture** | **0:** Majority of villi are > 3 crypt lengths long |
|  | **1:** Villi are ≤ 3 but > 1 crypt length long, with abnormality in ≤ 50% of mucosa. |
|  | **2:** Villi are ≤ 3 but > 1 crypt length long, with abnormality in > 50% of mucosa |
|  | **3:** Villi absent, or ≤ 1 crypt length long, with abnormality in ≤ 50% of mucosa |
|  | **4:** Villi absent, or ≤ 1 crypt length long, with abnormality in > 50% of mucosa |
| **Intramucosal Brunner’s glands** | **0:** Brunner glands are in submucosa, but are not observed above the muscularis mucosae |
|  | **1:** 1–2 foci, none involving > 5 crypt bases |
|  | **2:** 3–5 foci, none involving > 5 crypt bases |
|  | **3:**  > 5 foci, or any area of intramucosal Brunner glands involving > 5 crypt bases |
| **Goblet cells** | **0:** Normal goblet cell density (at least 1 goblet cell per 20 enterocytes) in all evaluable mucosal epithelial layer |
|  | **1:** Decreased goblet cells (< 1/20 enterocytes) in 1–25% of evaluable mucosal epithelium |
|  | **2:** Decreased goblet cells (< 1/20 enterocytes) in 26–50% of evaluable mucosal epithelium |
|  | **3:** Decreased goblet cells (< 1/20 enterocytes) in 51–75% of evaluable mucosal epithelium |
|  | **4:** Decreased goblet cells (< 1/20 enterocytes) in 76–100% of evaluable mucosal epithelium |
| **Paneth cells** | **0:** ≥ 5 Paneth cells/ crypt, on average |
|  | **1:** 2–4 Paneth cells/ crypt, on average |
|  | **2:** < 2 Paneth cell/crypt, involving ≤ 50% of crypt bases |
|  | **3:** < 2 Paneth cell/crypt, involving > 50% of crypt bases |

**From Kelly et. al.** (8)

**Supplemental Table 3: Protein and Transcript Correlations in Duodenal Tissue**

| **Model** | **Est.** | **95% CI** | **Intercept** | **n** | **Intraclass correlation coefficient (subject)** |
| --- | --- | --- | --- | --- | --- |
| *CD19* | 0.21 | 0.02, 0.41 | 4.1 | 132 | 0.48 |
| *CD3* | 0.08 | 0.01, 0.16 | 6.45 | 132 | 0.56 |
| *CD45* | 0.03 | -0.09, 0.15 | 6.94 | 108 | 0.34 |
| *CXCL10* | -0.09 | -0.21, 0.03 | 3.52 | 131 | 0.64 |
| *GZMB* | -0.08 | -0.2, 0.03 | 3.16 | 118 | 0.72 |
| *LCN2* | 0.38 | 0.26, 0.5 | 8.24 | 119 | 0.93 |
| *DEFA5* | -0.14 | -0.32, 0.05 | 10.77 | 130 | 0.57 |
| *MUC2* | -0.25 | -0.41, -0.09 | 8.47 | 132 | 0.55 |
| *REG1B* | 0.05 | -0.14, 0.25 | 4.97 | 131 | 0.76 |
| *SLC15A1* | 0.02 | -0.08, 0.13 | 9.16 | 111 | 0.93 |
| *SI* | -0.07 | -0.18, 0.04 | 10.86 | 132 | 0.63 |
| *DUOX2* | 0.04 | -0.05, 0.13 | 3.08 | 118 | 0.95 |

**Supplemental Figure 1. Enrollment flow chart by group**

Comparison

44

Comparison

39

BEECH

108

BEECH

28

BEECH

30

BEED

120

BEED

39

BEED

41

SEEM

63

SEEM

49

SEEM

49

Comparison

39

**Clinical Data Available and Verified**

**Tissue Transcriptomic Data Available**

**at Least one Duodenal H+E Slide of Sufficient Quality to Score**

**Supplemental Figure 1: Flow chart of Enrollment by Study Group.**


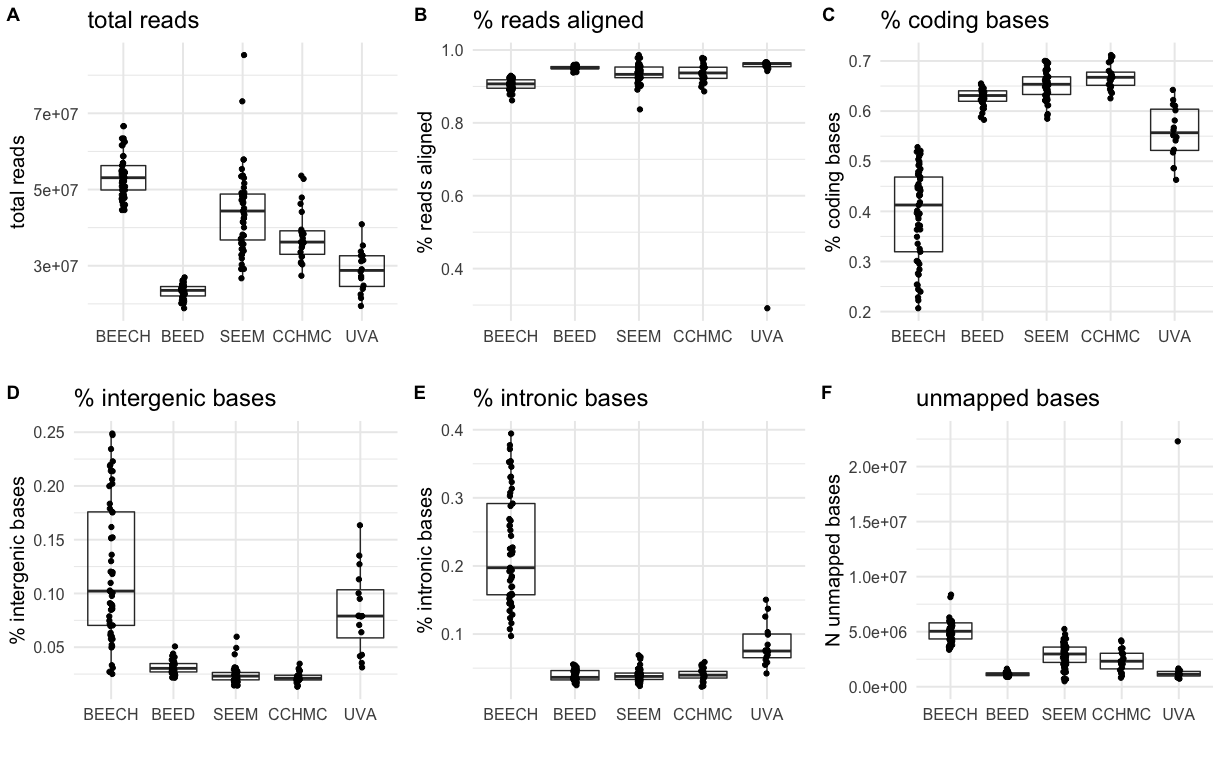


**Supplemental Figure 2: Sequencing Library Characteristics.** Quality control metrics for each sequencing library are shown by site. **A.** Total number of reads B. % reads that were aligned to sequences in the human genome **C.** % of reads that aligned to coding bases **D.** % of reads aligned to intergenic bases **E.** % of reads aligned to intronic bases **F.** The number of unmapped reads. Boxplots indicate the median, first quartile and third quartile in the data set with error bars indicating the minimum and maximum values.


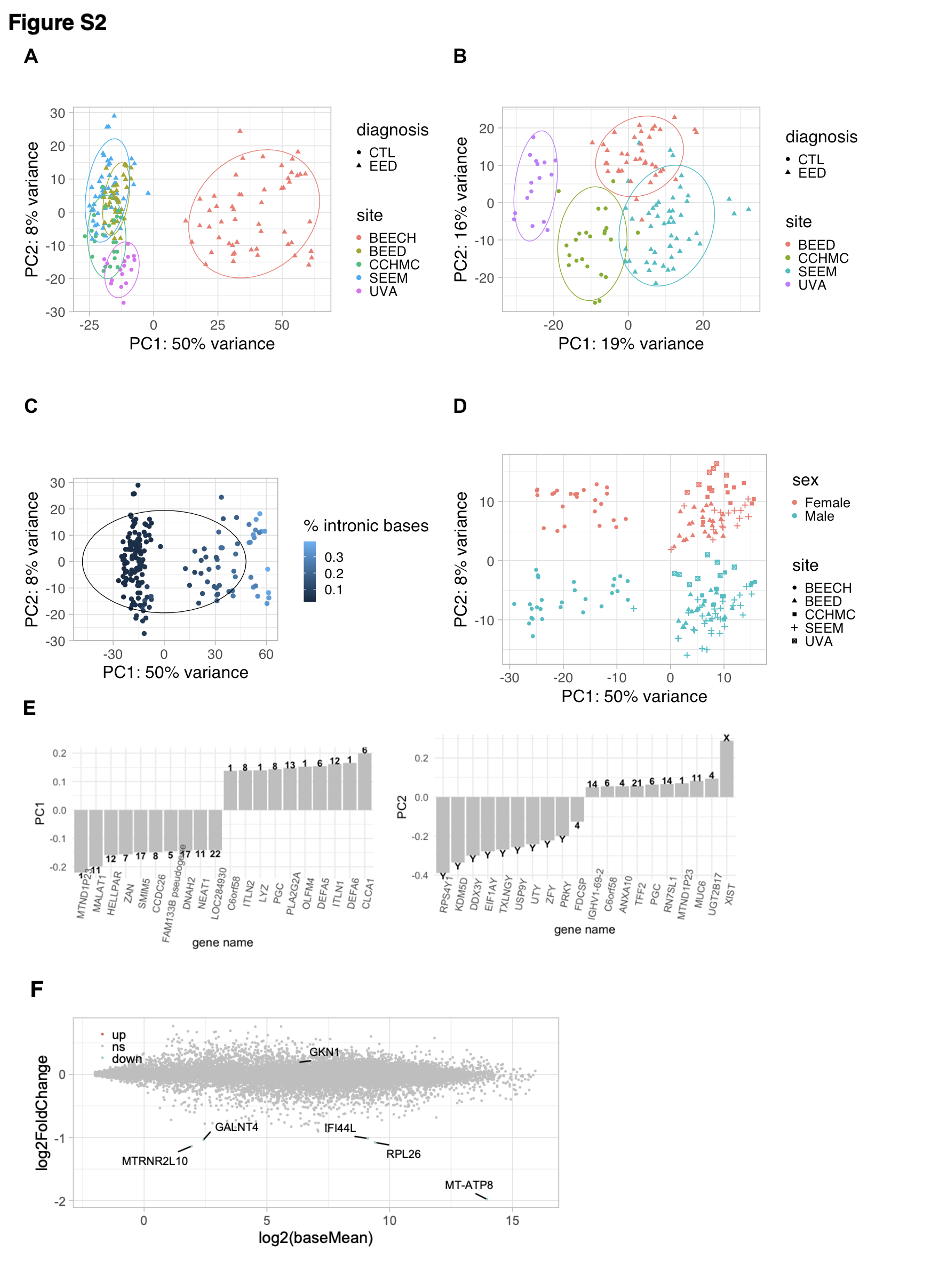


**Supplemental Figure 3: Effects of Covariates on Gene Expression in EED.** A. PCA plot of EED (triangle) and comparison (CTL) (circle) samples colored according to study center. B. PCA plot of EED (triangle) and comparison (CTL) (circle) samples colored according to site with BEECH samples excluded. C. PCA plot of all samples colored according to the % intronic bases. D. PCA plot of the top 100 most variable genes colored by sex, study center is indicated by symbol shape E. Gene loadings driving PC1 and PC2 in Figure 2D. Gene loadings are labeled according to their chromosomal location. F. Bland-Altman (MA) plot of genes that are differentially regulated by age in the comparison cohort. Genes that met the criteria for differential expression at the threshold of LFC >1 in either direction and *P*_adj_ <0.05 are labeled.


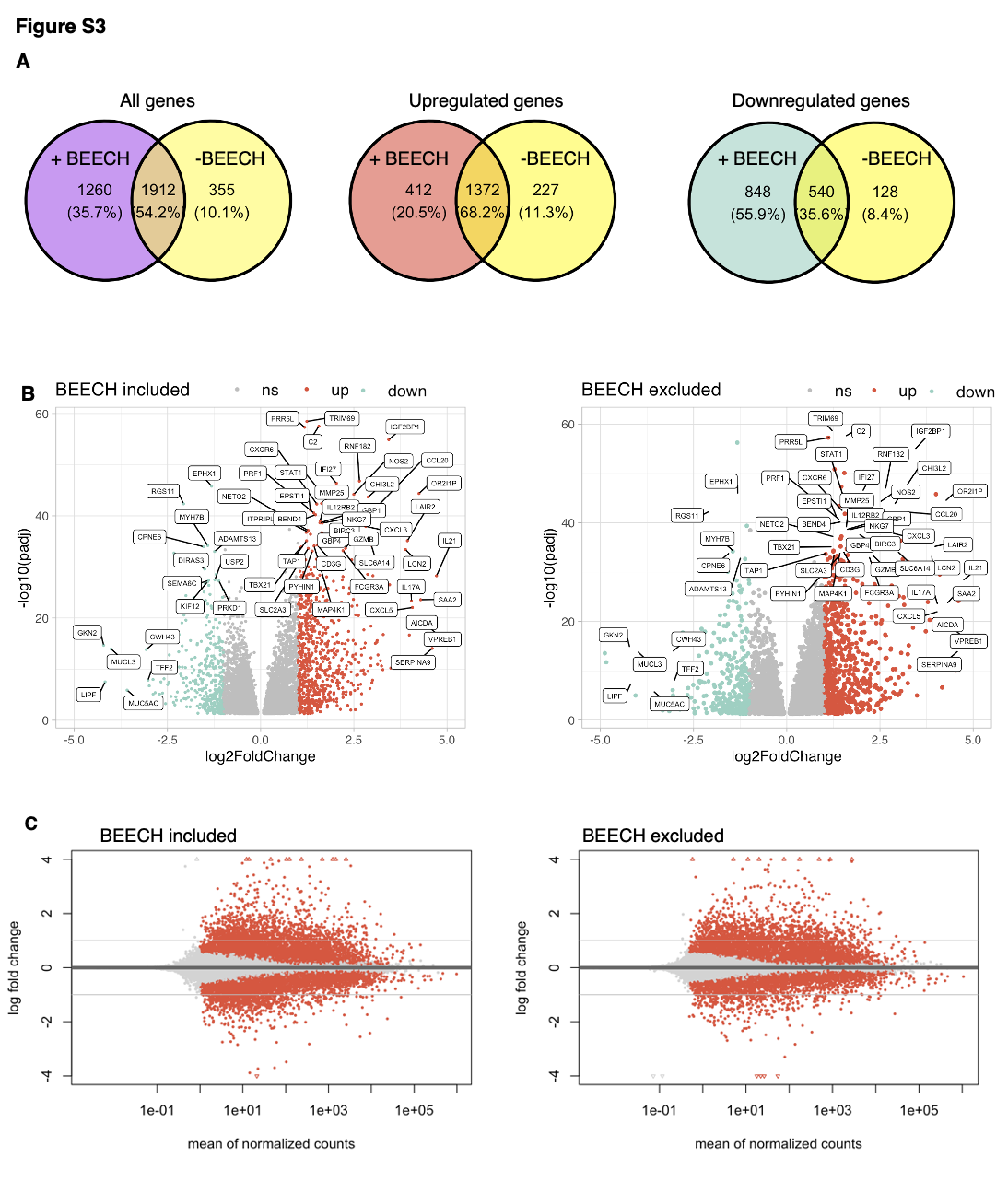


**Supplemental Figure 4**. **Sensitivity Analysis of Differential Expression** A. Overlap between the differentially expressed gene sets with and without BEECH samples. B. Volcano plots of differentially expressed genes with and without BEECH. C. Bland–Altman (MA) plot of differential expression with and without BEECH. Overall expression (X-axis) is shown relative to differential expression in EED (Y-axis).

**
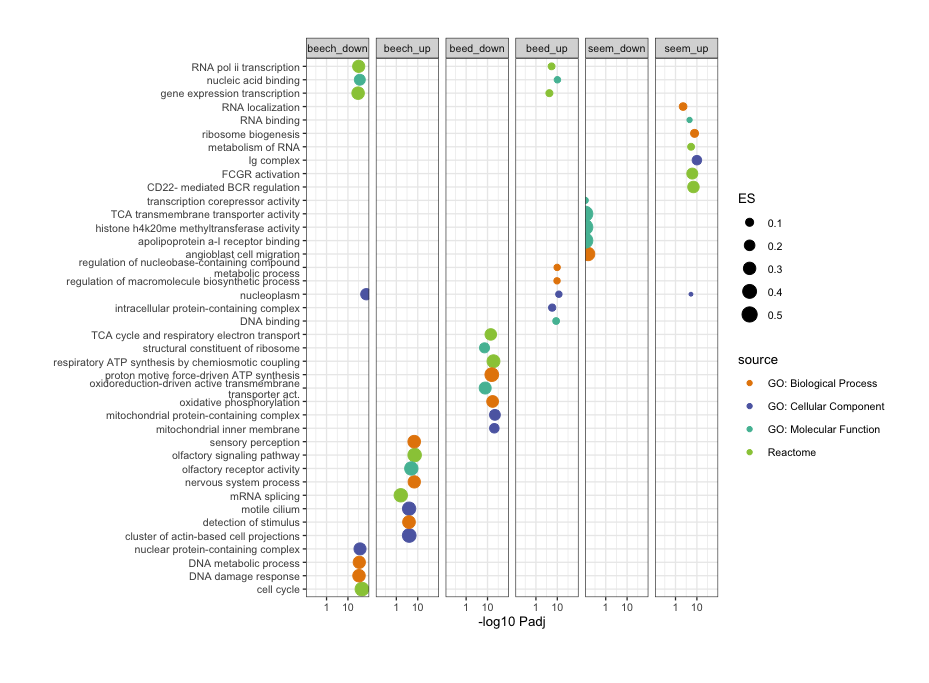
**

**D**

**Supplemental Figure 5**. **Intersite variability of EED** A. Bland–Altman (MA) plots of differentially expressed genes of each EED site relative to North American comparison group. The number of genes meeting the *P*_adj_ cutoff of <0.05 is shown. The multisite site analysis of all EED groups relative to North American comparison group (ALL) is included comparison B. The proportion of coding and non-coding genes identified in each geneset C. Venn diagram of overlap between the differentially expressed gene sets by site. D. Functional profiling of unique differentially expressed genes by site for overrepresented GO terms (CC: cellular component, MF: molecular function, BP: biological process), reactome biological pathways. Size corresponds to simple enrichment score (ES) (calculated as [# of genes in query geneset/# of genes in genome]).

**Supplemental Material References**

1. Y. Haberman, N.T. Iqbal, S. Ghandikota, I. Mallawaarachchi, T. Braun, P.J. Dexheimer, et al., Mucosal genomics implicate lymphocyte activation and lipid metabolism in refractory environmental enteric dysfunction, Gastroenterology 160 (2021) 2055–2071.e0, https://doi.org/10.1053/ j.gastro.2021.01.221.

2. B. Amadi, K. Zyambo, K. Chandwe, E. Besa, C. Mulenga, S. Mwakamui, et al., Adaptation of the small intestine to microbial enteropathogens in Zambian children with stunting, Nat. Microbiol. 6 (2021) 445–454, https://doi.org/ 10.1038/s41564-020-00849-w.

3. Love MI, Huber W, Anders S. Moderated estimation of fold change and dispersion for RNA-seq data with DESeq2. Genome Biol 2014;15:550.

4. Montgomery K, Gockley J, Han D. sageseqr: Identify differentially expressed genes counts data. [Internet]. 2022. Available from: https://sage-bionetworks.github.io/sageseqr, https://github.com/Sage-Bionetworks/sageseqr.

5. Stephens M. False discovery rates: a new deal. Biostatistics 2017;18:275–94.

6. Cunningham F, Allen JE, Allen J, Alvarez-Jarreta J, Amode MR, Armean IM, Austine-Orimoloye O, Azov AG, Barnes I, Bennett R, et al. Ensembl 2022. Nucleic Acids Res 2022;50:D988–95.

7. Raudvere U, Kolberg L, Kuzmin I, Arak T, Adler P, Peterson H, Vilo J. g:Profiler: a web server for functional enrichment analysis and conversions of gene lists (2019 update). Nucleic Acids Res 2019;47:W191–8.

8. P. Kelly, K. VanBuskirk, D. Coomes, S. Mouksassi, G. Smith, Z. Jamil, et al., Histopathology underlying environmental enteric dysfunction in a cohort study of undernourished children in Bangladesh, Pakistan, and Zambia compared with United States children, Am. J. Clin. Nutr. 120 (S1) (2024) S15–S30.

9. Z. Jamil, K. VanBuskirk, M. Mweetwa, S. Mouksassi, G Smith, T. Ahmed, et al. Anthropometry relationship with duodenal histological features of children with environmental enteric dysfunction: a multicenter cross-sectional study. Am. J. Clin. Nutr. 120 (S1) (2024) S64–S71.
